# Oxidized Low-Density Lipoproteins Trigger Hepatocellular Oxidative Stress with the Formation of Cholesteryl Ester Hydroperoxide-Enriched Lipid Droplets

**DOI:** 10.3390/ijms24054281

**Published:** 2023-02-21

**Authors:** Iku Sazaki, Toshihiro Sakurai, Arisa Yamahata, Sumire Mogi, Nao Inoue, Koutaro Ishida, Ami Kikkai, Hana Takeshita, Akiko Sakurai, Yuji Takahashi, Hitoshi Chiba, Shu-Ping Hui

**Affiliations:** 1 Faculty of Health Sciences, Hokkaido University, Sapporo 060-0812, Japan; 2 School of Medical Technology, Health Sciences University of Hokkaido, Sapporo 002-8072, Japan; 3 Department of Nutrition, Sapporo University of Health Sciences, Sapporo 007-0894, Japan

**Keywords:** LDL, non-alcoholic steatohepatitis, non-alcoholic fatty liver disease, liquid chromatography-mass spectrometry, lipidomics

## Abstract

Oxidized low-density lipoproteins (oxLDLs) induce oxidative stress in the liver tissue, leading to hepatic steatosis, inflammation, and fibrosis. Precise information on the role of oxLDL in this process is needed to establish strategies for the prevention and management of non-alcoholic fatty liver disease (NAFLD) and non-alcoholic steatohepatitis (NASH). Here, we report the effects of native LDL (nLDL) and oxLDL on lipid metabolism, lipid droplet formation, and gene expression in a human liver-derived C3A cell line. The results showed that nLDL induced lipid droplets enriched with cholesteryl ester (CE) and promoted triglyceride hydrolysis and inhibited oxidative degeneration of CE in association with the altered expression of *LIPE*, *FASN*, *SCD1*, *ATGL,* and *CAT* genes. In contrast, oxLDL showed a striking increase in lipid droplets enriched with CE hydroperoxides (CE-OOH) in association with the altered expression of *SREBP1*, *FASN*, and *DGAT1*. Phosphatidylcholine (PC)-OOH/PC was increased in oxLDL-supplemented cells as compared with other groups, suggesting that oxidative stress increased hepatocellular damage. Thus, intracellular lipid droplets enriched with CE-OOH appear to play a crucial role in NAFLD and NASH, triggered by oxLDL. We propose oxLDL as a novel therapeutic target and candidate biomarker for NAFLD and NASH.

## 1. Introduction

Non-alcoholic fatty liver disease (NAFLD), characterized by the accumulation of fat stored in liver cells, affects people who consume little to no alcohol [1]. These patients are referred to as having simple steatosis (SS). Simple steatosis can progress to non-alcoholic steatohepatitis (NASH) with hepatitis and liver fibrosis when additional oxidative and cytokine stress occurs [2]. Non-alcoholic steatohepatitis is irreversible and progresses to cirrhosis and hepatic carcinoma [3]. Simple steatosis and NASH are collectively defined as NAFLD, which is often observed in patients with metabolic disorders (such as obesity and type 2 diabetes) [4,5]. The global prevalence of NAFLD and NASH in the general population is estimated to be 10–35% and 3–5%, respectively [2]. In the USA, 34% of the general adult population (or at least 43 million adults) have NAFLD, and 12% have NASH [6,7]. Furthermore, an estimated 20% of patients with NASH develop cirrhosis, and NASH is projected to become the leading indication for liver transplantation in the United States [8,9]. Thus, it is important to prevent the progression of SS to NASH. Currently, the histopathological diagnosis of NASH requires invasive liver biopsies and is clinically problematic because of the lack of useful noninvasive blood testing methods. Therefore, elucidation of NASH pathogenesis is urgently required.

In addition to the fatty liver, NAFLD and NASH are also associated with dyslipidemia [10]. Particularly, patients with NASH have increased plasma oxidized low-density lipoproteins (oxLDL), which contain lipid hydroperoxides [11]. Lipid peroxidation is a leading factor in the development and progression of NASH [12]. Therefore, the oxLDL level is considered a risk factor for NASH.

Our laboratory previously reported a NASH mouse model that was fed a long-term high-fat diet and administered with oxLDL [13]. This suggests that oxLDL is one of the factors involved in the pathogenesis of NASH. Clarifying the association between oxLDL and lipid droplet formation in hepatocytes can contribute to the understanding of NASH pathogenesis. However, only a few studies have focused on oxLDL levels and hepatocytes. There have been no reports on the involvement of oxLDL compared with native low-density lipoproteins (nLDL) in the mechanism of lipid droplet formation, the components of lipid droplets, and the increase of oxidative stress in hepatocytes. Herein, we clarified that the addition of nLDL or oxLDL to human liver-derived C3A cells causes fat accumulation and analyzed the lipid components using liquid chromatography-tandem mass spectrometry (LC-MS/MS) and transcriptional expression analyses to better understand lipid metabolic changes in the cells.

## 2. Results

### 2.1. Lipidomic Analysis in LDL

We analyzed the profiles of cholesteryl ester (CE), triacylglycerol (TG), and their hydroperoxides in nLDL and oxLDL (which were added to C3A cells) because the major lipid components in lipid droplets are CE and TG [14]. Six CE molecular species were detected in the LDL particles (Supplementary Figure S1). All molecular species except CE 16:0 decreased with increasing oxidation time. The behavior of the sum of CE was consistent with that of each CE molecular species (Supplementary Figure S2). CE 20:5 was not detected in oxLDL (24 h). In the detection of CE hydroperoxide (CE-OOH), only CE-OOH 18:2, which increased considerably only in oxLDL (2 h), was detected (Supplementary Figure S3).

Furthermore, 41 TG molecular species were detected in the LDL particles (Supplementary Figure S4). Similar to CE, almost all TG molecular species decreased with longer oxidation times. The behavior of the sum of TG was consistent with that of each TG molecular species (Supplementary Figure S5). In the detection of TG hydroperoxide (TG-OOH), 12 TG-OOH molecules were detected (Supplementary Figure S6). Similar to CE-OOH, the sum of the TG-OOH levels was considerably high in oxLDL (2 h) under all conditions (Supplementary Figure S7).

Regarding phosphatidylcholine (PC), 22 PC and 10 PC hydroperoxides (PC-OOH) were detected in LDL (Supplementary Figures S8–S11). Additionally, the sum of PC-OOH was the highest in oxLDL (2 h).

Taken together, oxLDL (2 h) was enriched in CE-OOH, TG-OOH, and PC-OOH compared with LDL at other oxidation times (8, 24 h).

### 2.2. Cell Toxicity Test

**Figure 1:**
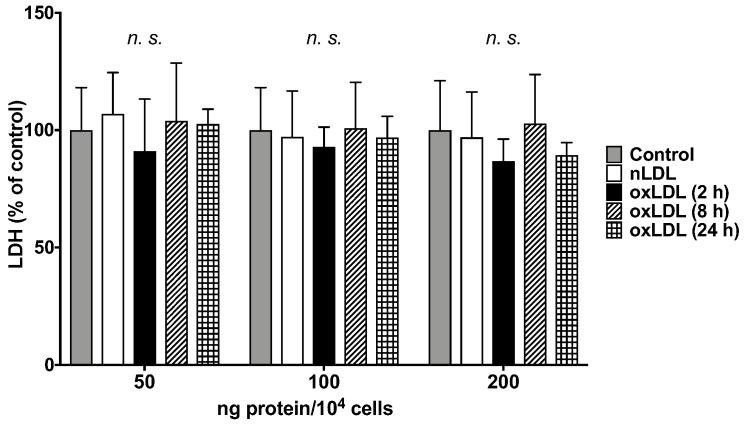
Cell toxicity test of C3A cells at 24 h after adding nLDL or oxLDL. Cell toxicity in the control group phosphate-buffered saline (PBS) was set as 100%. Results are expressed as mean ± standard deviation. *n* = 6. One-way analysis of variance (ANOVA) with Dunnett’s multiple comparisons test, *n.s.* not significant.

To evaluate the toxicity of LDL in C3A cells, lactate dehydrogenase (LDH) in the culture supernatant was analyzed (Figure 1). Under the present ranges of added LDL concentrations, no reduction in cell toxicity was observed in nLDL and any oxLDL groups (2, 8, and 24 h) compared with the control group at 50, 100, and 200 ng protein of LDL/10^4^ cells. Thus, nLDL/oxLDL concentrations of 200 ng protein/10^4^ cells were used to stimulate nLDL/oxLDL in this study.

### 2.3. Fluorescence Imaging of LDL-Induced Lipid Droplets

**Figure 2:**
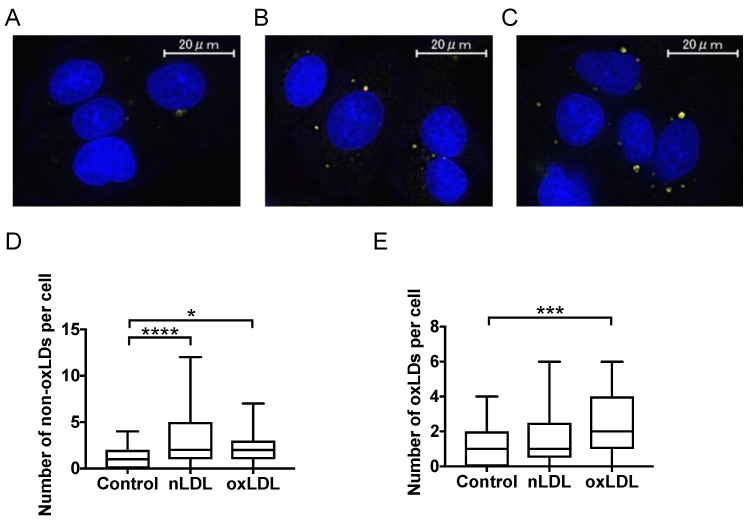
Evaluation of accumulation of neutral lipids and lipid hydroperoxides using fluorescence imaging. (**A**–**C**) Lipid droplets with neutral lipids and lipid hydroperoxides formed by C3A cells supplemented with (**A**) PBS, (**B**) 2.0 ng protein/µL nLDL, and (**C**) 2.0 ng protein/µL oxLDL (100×). (**D**) Number of non-oxidized lipid droplets (non-oxLDs) per cell analyzed using fluorescence imaging. (**E**) Number of oxidized lipid droplets (oxLDs) per cell analyzed using fluorescence imaging. Results were expressed as a box plot (*n* = 53–84). One-way ANOVA with Kruskal–Wallis test, * *p* < 0.05, *** *p* < 0.001, **** *p* < 0.0001.

According to a previous report [15], fluorescence imaging was used for observing nLDL- or oxLDL (2 h)-induced lipid droplets, nuclei (blue), and the accumulation of neutral lipids (red) and lipid hydroperoxides (green) (Figure 2A–C). The locations where neutral lipids and lipid hydroperoxides overlapped are shown in yellow. As a result, their behaviors were different from each other. Native LDL increased the number of non-oxidized lipid droplets (non-oxLDs) and not oxidized lipid droplets (oxLDs), whereas oxLDL increased the number of oxLDs and non-oxLDs compared with the control (Figure 2D,E).

### 2.4. Lipidomic Analysis in LDL-Supplemented C3A Cells

**Figure 3:**
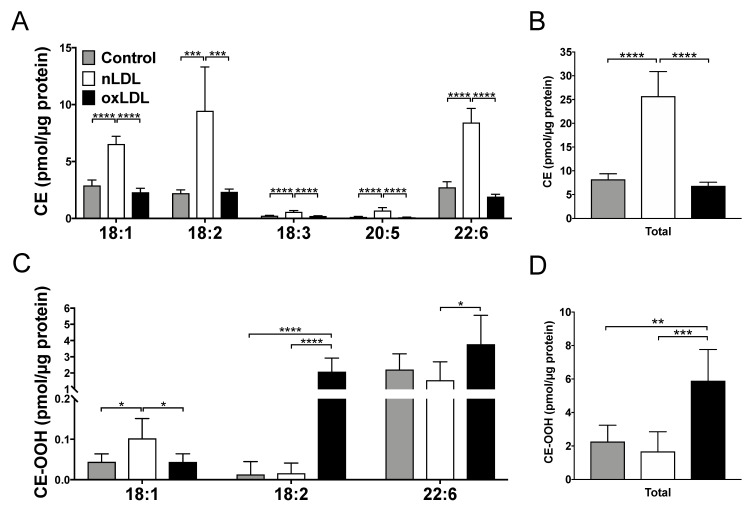
Comparison of CE and CE-OOH species in the LDL-supplemented C3A cells detected using Orbitrap LC-MS/MS. (**A**) CE species. (**B**) The sum of CEs detected in this experiment. (**C**) The CE-OOH species. (**D**) The sum of CE-OOHs detected in this experiment. Results are shown as mean ± standard deviation. *n* = 6. One-way ANOVA with Tukey’s multiple comparisons test, * *p* < 0.05, ** *p* < 0.01, *** *p* < 0.001, **** *p* < 0.0001.

Five CE molecular species were detected in LDL-supplemented cells (Figure 3A). The levels of all molecular species were considerably high in the nLDL group. Similarly, the sum of CE was also increased considerably in the nLDL group (Figure 3B).

Three types of CE-OOH molecules (CE-OOH 18:1, CE-OOH 18:2, and CE-OOH 22:6) were also detected in LDL-treated cells (Figure 3C). The sum of CE-OOH increased considerably only in oxLDL (2 h)-treated cells (Figure 3D).

**Figure 4:**
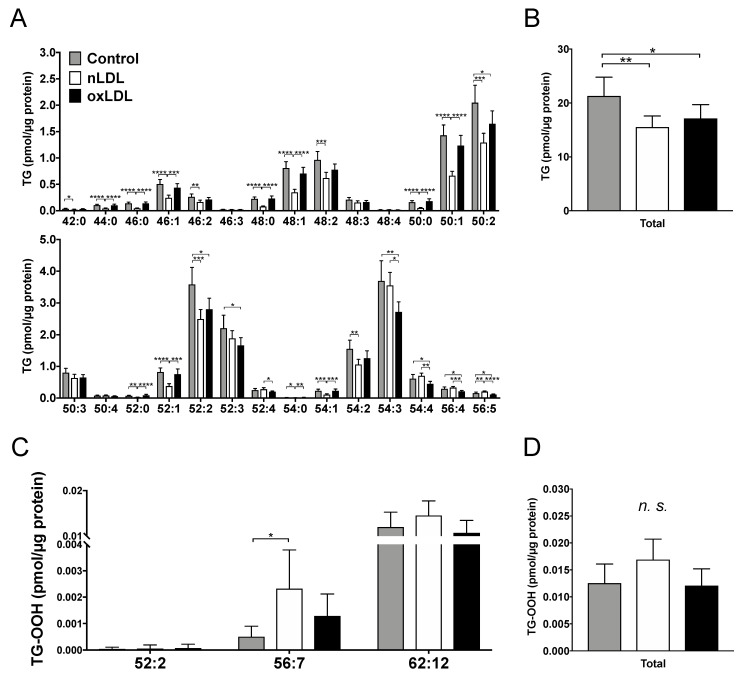
Comparison of TG and TG-OOH species in the LDL-supplemented C3A cells detected using Orbitrap LC-MS/MS. (**A**) TG species. (**B**) The sum of TGs detected in this experiment. (**C**) TG-OOH species. (**D**) The sum of TG-OOHs detected in this experiment. Results are shown as mean ± standard deviation. *n* = 6. One-way ANOVA with Tukey’s multiple comparisons test, * *p* < 0.05, ** *p* < 0.01, *** *p* < 0.001, **** *p* < 0.0001, *n.s.* not significant.

Regarding the TG profile, 28 TG molecular species were detected in the LDL-supplemented cells (Figure 4A). In addition, 17 TG molecular species showed a considerable decrease in the nLDL group compared with the control. Furthermore, seven TG molecular species were decreased considerably in the oxLDL group compared with the control. The sum of the TG molecular species was reduced considerably in the nLDL and oxLDL groups compared with that in the control group (Figure 4B). Furthermore, three types of TG-OOH molecules (TG-OOH 52:2, TG-OOH 56:7, and TG-OOH 62:12) were detected in LDL-treated cells (Figure 4C). Overall, there was no notable difference in the sum of TG-OOH levels among the three groups (Figure 4D).

Seventeen PC molecular species were detected in LDL-supplemented cells (Supplementary Figure S12A). The levels of all molecular species and the sum of PC were considerably higher in the nLDL-supplemented cells than in the oxLDL-supplemented cells and the control (Supplementary Figure S12B).

**Figure 5:**
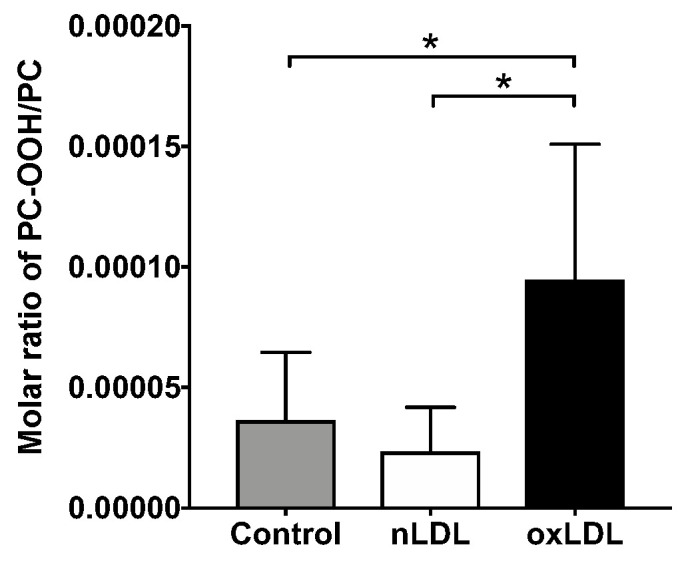
The molar ratio of PC-OOH/PC. Results are shown as mean ± standard deviation. *n* = 5–6. One-way ANOVA with Tukey’s multiple comparisons test, * *p* < 0.05.

Two types of PC-OOH molecules (36:5 and 36:6) were also detected in LDL-supplemented cells (Supplementary Figure S12C). Although there was no notable difference, the sum of PC-OOH showed an increasing trend in the oxLDL-supplemented cells (Con. vs. oxLDL, *p* = 0.093; nLDL vs. oxLDL, *p* = 0.103) (Supplementary Figure S12D). The sum of PC-OOH/PC, used as an index of cellular oxidative stress, increased considerably only in oxLDL-supplemented cells (Figure 5).

### 2.5. Expression of Genes in Lipid Metabolism

**Figure 6:**
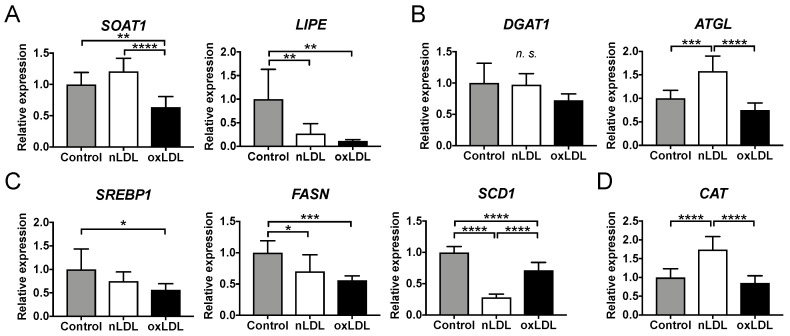
Expression of genes related to lipid metabolism in the LDL-supplemented C3A cells detected using real-time PCR. (**A**) Biosynthesis and degradation of CE: *SOAT1* and *LIPE*; (**B**) Biosynthesis and degradation of TG: *DGAT1* and *ATGL*; (**C**) Biosynthesis marker of fatty acids: *SREBP1*, *FASN*, and *SCD1*; (**D**) Antioxidants: *CAT*. Results are shown as the relative expression levels compared to the control (PBS), which was set to 1.0. *n* = 6–8. One-way ANOVA with Tukey’s multiple comparisons test, * *p* < 0.05, ** *p* < 0.01, *** *p* < 0.001, **** *p* < 0.0001, *n.s.* not significant.

To investigate the transcriptional changes in lipid metabolism in LDL-supplemented C3A cells, real-time polymerase chain reaction (PCR) was performed under the same conditions as the LC-MS/MS experiments. The expression level of sterol O-acyltransferase 1 (*SOAT1*), a gene associated with CE biosynthesis, was reduced considerably in the oxLDL group compared with that in the control and nLDL groups (Figure 6A). The expression of lipase E, a hormone-sensitive-type (*LIPE*) gene associated with the degradation of CE, was markedly reduced in the nLDL- and oxLDL-supplemented cells compared with that in the control (Figure 6A). No considerable differences were observed in the expression of diacylglycerol O-acyltransferase 1 (*DGAT1*), associated with TG biosynthesis (Figure 6B). The expression level of adipose triglyceride lipase (*ATGL*), a gene associated with TG degradation, was considerably increased in the nLDL group only (Figure 6B).

The expression level of sterol regulatory element-binding protein 1 (*SREBP1*), a factor that regulates fatty acid biosynthesis, was decreased considerably in the oxLDL group compared with that in the control group (Figure 6C). The expression level of fatty acid synthase (*FASN*) was considerably reduced in the nLDL and oxLDL groups compared with that in the control group (Figure 6C). The expression of stearoyl-CoA desaturase (*SCD1*) for fatty acid unsaturation was considerably reduced in the oxLDL group compared with that in the control group and notably reduced in the nLDL group (Figure 6C). The expression of catalase (*CAT*), a hepatic antioxidant enzyme, was considerably increased in the nLDL group compared with that in the control and oxLDL groups (Figure 6D).

**Figure 7:**
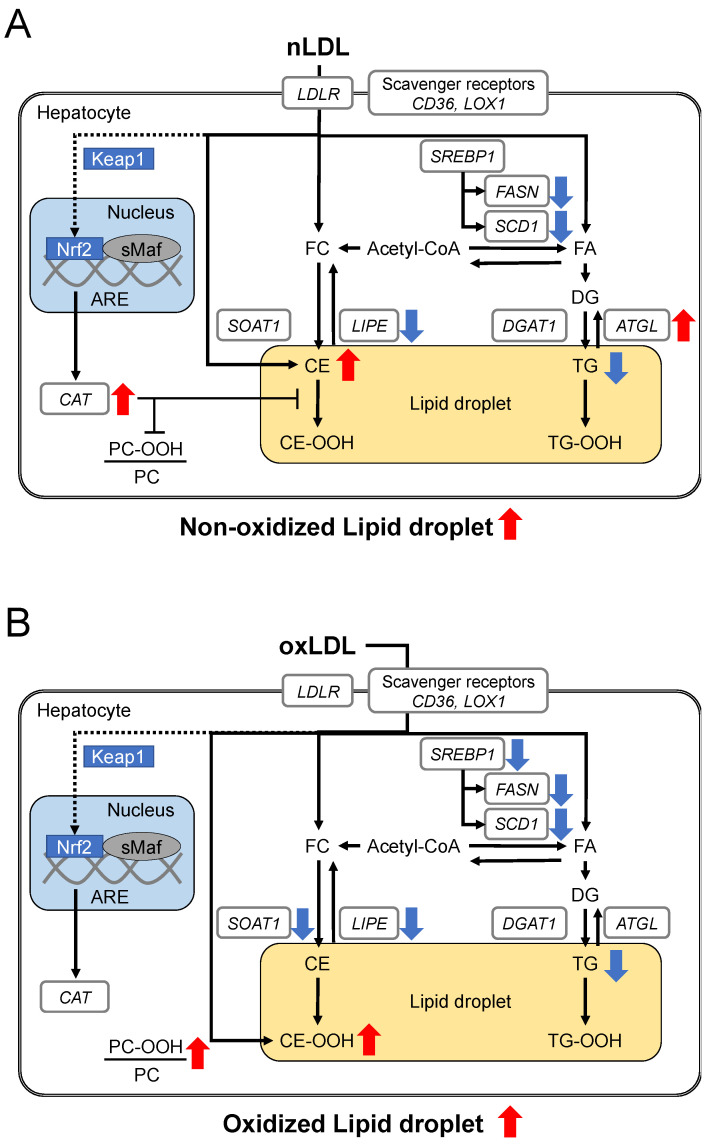
The hypothesized mechanism of lipid metabolism changes in the LDL-supplemented C3A cells. (**A**) Changes in lipid metabolism in the nLDL-supplemented C3A cells. nLDL decreased the expression of genes involved in CE lipolysis (*LIPE*), which may be associated with accumulated CE and increased lipid droplets. nLDL increased the expression of genes involved in fatty acid synthesis (*FASN* and *SCD1*) and TG hydrolysis (*ATGL*). Additionally, nLDL decreased some TG molecular species. Moreover, nLDL increased the expression of an antioxidative enzyme, *CAT*. Taken together, nLDL might exert protective effects on the hepatocytes by increasing TG hydrolysis and antioxidant enzyme expression. (**B**) Changes in lipid metabolism in the oxLDL-supplemented C3A cells. oxLDL caused downregulation of genes involved in CE metabolism (*LIPE* and *SOAT1*) and increased lipid hydroperoxide-containing lipid droplets, CE-OOH and PC-OOH/PC. oxLDL also decreased the expression of genes involved in fatty acid synthesis (*SREBP1*, *FASN*, and *SCD1*) and some TG.

From the above results, the lipid metabolic changes in nLDL- (Figure 7A) and oxLDL-supplemented C3A cells (Figure 7B) are summarized.

## 3. Discussion

The degree of oxidation varies among oxLDLs in plasma [16]; thus, oxLDL is a heterogeneous particle. Lipid components in LDL become hydroperoxides (-OOH) under early oxidative conditions and aldehydes (-CHO) with increasing degrees of oxidation [17,18]. Mild oxLDL reflects the physiological form of oxLDL and contains lipid hydroperoxides [19]; thus, mild oxLDL is toxic to the body. To determine the optimal oxidative conditions with high levels of lipid hydroperoxides, oxLDL was prepared using different oxidation times (0–24 h). The present experiments showed that the sum of CE decreased with increasing LDL oxidation time and that LDL oxidized for 2 h contained the highest levels of CE-OOH (Supplementary Figures S2 and S3). This suggests that CE may have been reduced by oxidative denaturation and transformed into CE-OOH in LDL, which was oxidized for 2 h. Further oxidation (8 and 24 h) resulted in the reduction of CE-OOH species (Supplementary Figure S3), which may subsequently produce aldehydes and other oxidative compounds. Similar to CE, the TG and PC levels decreased with increasing oxidation time (Supplementary Figures S4, S5, S8 and S9). TG-OOH and PC-OOH were the most abundant in LDL oxidized for 2 h (Supplementary Figures S6, S7, S10 and S11). Based on these results, oxLDL (2 h) with increased CE-OOH, TG-OOH, and PC-OOH was adopted as mildly oxidized LDL for the stimulation condition.

Fluorescence imaging analysis showed that both nLDL and oxLDL accumulated neutral lipids in hepatocytes, suggesting that nLDL or oxLDL was incorporated into the cells and excess lipids were stored in lipid droplets. Furthermore, oxLDL-supplemented cells were observed to have lipid hydroperoxides overlapping with neutral lipids as a reference [15]. This suggests that lipid hydroperoxides of added oxLDL may have accumulated in lipid droplets. CE-OOH levels in the liver tissue of patients with NASH are elevated [20], and the presence of lipid hydroperoxides (-OOH) in hepatocytes is closely associated with NASH [21]. Thus, oxLDL uptake may enhance oxidative stress in the hepatocytes.

Native LDL is taken up by hepatocytes via LDLR [22] and oxLDL via scavenger receptors, such as CD36 and LOX1 [23,24]. Simultaneous analysis of lipids in hepatocytes revealed that CE increased in the nLDL group. Native LDL is a CE-rich lipoprotein (Supplementary Figure S2). The lipid droplets that were stained in the fluorescence microscopy experiment can be derived from the CE in the incorporated nLDL (Figure 2). CE taken into cells can be degraded by hydrolysis [25,26], or CE synthesis can be suppressed by the downregulation of *SOAT1* (a gene associated with CE biosynthesis) [27]. In the present study, the expression level of *SOAT1* remained unchanged in nLDL-supplemented cells. In contrast, the expression level of *LIPE* (a gene involved in CE degradation) showed a distinct decrease (Figure 6A). Therefore, CE degradation can be suppressed, and subsequent CE accumulation occurs in hepatocytes. In contrast, CE was at the same level in the oxLDL group as in the control group (Figure 3A,B). This could be reasonable because oxLDL was markedly poor in CE owing to oxidative modifications of CE. The increase in CE-OOH in oxLDL-supplemented cells was smaller than the increase of CE in nLDL-supplemented cells. This might indicate that CE-OOH reacted with numerous other oxidative species (e.g., CE-CHO) that were not targeted in this study.

CE-OOH 18:2 and CE-OOH 22:6 were increased only in oxLDL-supplemented cells (Figure 3C), suggesting a state of increased intracellular oxidative stress. The increase in CE-OOH 18:2 levels in the cells implied that oxLDL (2 h) was rich in CE-OOH 18:2 (Supplementary Figure S3). These acyl chains are polyunsaturated fatty acids (PUFAs). Because PUFAs are susceptible to oxidation, CE with PUFA may be oxidized in oxLDL-supplemented cells. This increase was consistent with the detection of more lipid hydroperoxide using fluorescence staining (Figure 2) and was likely to be attributed to CE-OOH in oxLDL. In contrast, nLDL-supplemented cells showed no increase in CE-OOH (Figure 3), which can be attributed to less CE-OOH in nLDL-supplemented cells. Thus, hepatic antioxidant enzymes may exert inhibitory effects on the excessive oxidation of CE incorporated into the cells. The present transcriptional study revealed an increased expression of the antioxidant enzyme-related gene *CAT* in nLDL-supplemented cells (Figure 6D). *CAT* is a family of antioxidant enzymes induced by the activation of the Keap1-Nrf2 pathway [28]. It is most abundant in the liver, kidneys, and erythrocytes and is responsible for degrading most of the hydrogen peroxide [29]. Hydrogen peroxide generates lipid hydroperoxides. Thus, the induction of *CAT* could eliminate CE-OOH.

We predicted that TG derived from LDL might accumulate via LDL uptake. However, TG levels decreased in both nLDL- and oxLDL-treated cells (Figure 4A,B). An increase in *ATGL* (a gene involved in TG hydrolysis) promotes a decrease in TG accumulation [30]. Thus, in nLDL, the high expression level of *ATGL* in nLDL-supplemented cells may have promoted TG degradation and prevented TG accumulation in hepatocytes. However, the effects on the reduction of TG species were weaker in oxLDL-supplemented cells than in nLDL-supplemented cells (Figure 4A,B). This might be due to fewer TG species in the added oxLDL (Supplementary Figures S4 and S5) or few changes in TG species due to little induction of *ATGL* (Figure 4A and Figure 6B).

The sum of TG-OOH in the cells did not change among the three groups (Figure 4D), despite the addition of oxLDL, including high levels of TG-OOH. Because the amount of TG-OOH was smaller than that of CE-OOH, even in oxLDL (Supplementary Figures S3 and S7), the changes in the cells caused by TG-OOH of oxLDL may have been neglected.

PC-OOH is a primary peroxidative lipid that has been used to monitor hepatocellular damage by lipid peroxidation [31]. Liver PC-OOH levels were higher in NASH model mice than in control mice [21]. Furthermore, PC-OOH/PC has been used as an index of cellular oxidative damage [32]. Thus, we analyzed the PC-OOH and PC-OOH/PC using LC-MS/MS. We found that PC-OOH/PC was increased in oxLDL-supplemented cells compared with that in other groups, which suggests hepatocellular damage due to oxidative stress (Figure 5). In contrast, PC-OOH/PC was unchanged in nLDL, which suggests a protective effect of *CAT* against oxidation.

In the nLDL and oxLDL groups, the expression levels of genes associated with the synthetic pathway of fatty acids induced by acetyl-CoA were suppressed despite the formation of lipid droplets (Figure 6C). This was consistent with previous reports on mice with fatty livers [33]. This might indicate negative feedback against the accumulation of excessive lipids, possibly suppressing TG accumulation.

## 4. Materials and Methods

### 4.1. Separation of Total Lipoproteins

Blood samples were obtained from healthy participants after overnight fasting. Serum samples were separated by centrifugation at 2200× *g* for 10 min at 4 °C using a CE16RX (Hitachi Koki Co., Ltd., Tokyo, Japan). As previously reported, total lipoproteins were separated by ultracentrifugation [34]. Briefly, 2.0 mL of serum was adjusted to a density of 1.225 kg/L using potassium bromide (Fujifilm Wako Pure Chemical Corporation, Osaka, Japan) and mixed with 6.0 mL of a specific density solution (density = 1.225). The mixed solution was then ultracentrifuged at 50,000 rpm for 20 h at 4 °C in an Optima MAX Ultracentrifuge (Beckman Coulter Inc., Brea, CA, USA) with a near-vertical rotor MLN-80 (Beckman Coulter Inc., Brea, CA, USA). The total lipoprotein fraction was collected from the top layer.

### 4.2. Separation of LDL Fraction

Gel filtration chromatography was performed to separate LDL and other lipoproteins (very low-density lipoprotein and high-density lipoprotein) [34]. The total lipoprotein fraction was injected into a high-performance liquid chromatograph (HPLC, Shimadzu Corp., Tokyo, Japan) equipped with a Superose 6 column (GE Healthcare, Little Chalfont, UK). The lipoproteins were then eluted with 50 mM phosphate-buffered saline (PBS) (pH 7.4) at a rate of 0.5 mL/min and monitored at OD 280 nm. The LDL fractions were collected at an elution time of 21–27 min. The protein concentrations of these fractions were determined using the Lowry method [35].

### 4.3. Oxidization of LDL

As indicated in previous reports [36], LDL fractions were diluted to a protein concentration of 0.2 mg/mL with phosphate buffer (50 mM PBS, pH 7.4); copper sulfate was added to a final concentration of 0.06 mM and incubated for 2, 8, and 24 h in a thermostatic chamber at 37 °C. Oxidation was stopped by adding ethylenediaminetetraacetic acid (EDTA) to a final concentration of 1.0 mM. To prevent oxidation due to residual copper ions, the solvent was replaced with phosphate buffer (50 mM PBS, pH 7.4) using a 100 kDa filter (Merck Millipore Ltd., Cork, Ireland) [37]. Oxidized LDL solutions were diluted to a protein concentration of 0.2 mg/mL and stored at 4 °C until immediately before use. nLDL was added to equal volumes of water instead of copper sulfate. As with oxLDL, EDTA was added and stored at 4 °C until use.

### 4.4. Lipidomics Using LC/MS

Lipid extraction from each LDL was performed following the procedure described by Folch et al. [38] and Hui et al. [36] (*n* = 4 for each group). nLDL or oxLDL (600 µL) was added to 1.4 mL of distilled water and stirred (3500 rpm, 1 min) using a Multi-Speed Vortex (BIOSAN Ltd., Riga, Latvia). The mixture was transferred to a screw-tip test tube. Internal standards (IS, SPLASH™LIPIDOMIX^®^ Quantitative Mass Spec Internal Standard, Avanti Polar Lipids, Inc., Alabaster, AL, USA) were diluted 50-fold with methanol. Next, 100 µL of diluted IS, 300 µL of methanol, and 2 mL of chloroform were added sequentially, stirred, and centrifuged (2200× *g*, 4 °C, and 10 min). The lower chloroform layer was collected. Then, 2 mL of chloroform was added to the remaining upper layer, the above collection procedure was repeated, and the lower layer was collected again. The solution was then dried using an evaporator (centrifugal concentrator CC-105; Tomy Industries, Tokyo, Japan). After drying, 300 µL of methanol was added, and the mixture was stirred to collect the total volume. The samples were centrifuged (18,800× *g*, 4 °C, 10 min), and the supernatant was collected. The samples were stored at −80 °C until immediately before measurement.

For the simultaneous analysis of lipids using Orbitrap LC-MS, cells stimulated with LDL were collected (*n* = 6 for each group). C3A cell suspensions (10% fetal bovine serum, FBS) were seeded in 24-well plates at 2.0 × 10^5^ cells/mL and 1 mL/well, and preincubation and stimulation were performed under the same conditions as for fluorescence staining. The cells were then washed once with PBS. PBS (500 µL) was added, and the cells were collected using a scraper. Fifty microliters of this solution were used to determine the protein concentration of the cells. The cells in the remaining 450 µL were precipitated by centrifugation, and the supernatant was discarded. Next, the cells were washed with PBS, centrifuged again, and the supernatant discarded. The precipitated cell mass was used as a sample and stored at −80 °C until immediately before lipid extraction from the cell mass.

Lipids were extracted from each cell following the procedure described by Hara et al. [39] (*n* = 6 for each group). Diluted IS (200 µL) and chloroform (400 µL) were added to the collected cell mass and agitated using a Multi-Speed Vortex (BIOSAN, Ltd, Riga, Latvia.). The mixture was then centrifuged (Himac CE15R, Hitachi Koki Co., Ltd., Tokyo, Japan, 18,800× *g*, 4 °C, 10 min). The supernatant was collected, and the solution was allowed to dry using a centrifugal concentrator (CC-105, Tomy Industries, Tokyo, Japan). Next, 400 µL chloroform was added to the sample before transferring the supernatant and stirring under the same conditions. It was centrifuged, and the supernatant was collected into a sample that had just been allowed to dry. The solution was allowed to dry again, 100 µL of methanol was added, and the mixture was stirred using a Multi-Speed Vortex (BIOSAN Ltd., Riga, Latvia). The mixture was centrifuged, and the supernatant was collected. The samples were stored at −80 °C until immediately before measurement.

Orbitrap LC-MS/MS was used for the simultaneous analysis of lipids in LDL and liver culture cell C3A, based on previous reports [40]. The LC section was performed on a Shimadzu Prominence HPLC system (Shimadzu Corp., Kyoto, Japan), and the analytical column was an Atlantis T3 Column (C18, 2.1 × 150 nm, and 3 µm; Waters Corp., Milford, CT, USA). The column temperature was 40 °C, and the sample injection volume was 10 µL. A gradient elution method was used, and the mobile phase consisted of 5 mM ammonium acetate (A), isopropanol (B), and methanol (C). The flow rate was 0.2 mL/min; the MS section was an LTQ Orbitrap mass spectrometer (Thermo Fisher Scientific Inc., Waltham, MA, USA). Depending on the target molecule, the analysis was performed in the electrospray ionization (ESI)-positive ion mode (Supplementary Table S1). Peak areas were calculated using Xcalibur 2.2 (Thermo Fisher Scientific Inc., Waltham, MA, USA). Based on published data, we identified the peak using the LIPIDMAPS database [21]. The identified species were annotated as class abbreviations: lipid class, total number of carbons in fatty acid moieties:total number of double bonds in the acyl chains (e.g., TG 52:2) (Supplementary Tables S2–S5).

When calculating the lipid concentrations in LDL, the peak areas of the target molecules were corrected using the peak area of the IS. When calculating intracellular lipid concentrations, the peak area was corrected using the peak area of the IS and protein concentration in the hepatocytes. CE, TG, PC, and their peroxides (CE-OOH, TG-OOH, and PC-OOH) were analyzed.

### 4.5. Cell Culture and Toxicity Test

Human liver-derived strain C3A (ATCC) cells were passaged and cultured in MEM supplemented with fetal bovine serum (FBS, Thermo Fisher Scientific Inc., Waltham, MA, USA, final concentration of 10%), penicillin-streptomycin (Thermo Fisher Scientific Inc., Waltham, MA, USA, final concentration 1%), and GlutaMAX supplement (GlutaMAX, Thermo Fisher Scientific Inc., Waltham, MA, USA, final concentration of 1%) at 37 °C and 5% CO_2_.

To confirm the hepatotoxicity of LDL, a cell toxicity test was performed (*n* = 6 for each group): 1.0 × 10^4^ cells/mL of C3A (10% FBS-containing medium) were seeded in 96-well plates at 100 µL/well and pre-incubated at 37 °C and 5% CO_2_ for 24 h. For the preparation of stimulants, the supernatant was mixed with Clear MEM (0% FBS, Thermo Fisher Scientific Inc., Waltham, MA, USA) with LDL solution adjusted to 50, 100, and 200 ng protein per 1 × 10^4^ cells in the nLDL and oxLDL groups. An equal volume of PBS was used instead of LDL for the control group. The supernatant was then replaced with stimulants (100 µL/well), and cells were stimulated at 37 °C and 5% CO_2_ for 22 h. At the end of stimulation, the supernatant was collected. LDH was measured for toxicity testing according to the manufacturer’s instructions (Takara Bio Inc., Shiga, Japan). Absorbance at 490 nm was measured using a microplate reader (xMark™ Microplate Spectrophotometer, Bio-Rad Laboratories, Inc., Hercules, CA, USA). The values are shown as the percentage of cell toxicity, with the control set to 100%.

### 4.6. Fluorescence Microscopy

With reference to Tsukui et al. [15], fluorescence staining was performed to quantify the lipid droplets observed when LDL was added. C3A cell suspensions (10% FBS) were seeded with 2.0 × 10^5^ cells of C3A in glass-bottomed dishes and pre-cultured for 24 h at 37 °C and 5% CO_2_. The cells were washed once with PBS (Fujifilm Wako Pure Chemical Corporation, Osaka, Japan). For the preparation of the stimulant medium, Clear MEM (0% FBS) was mixed with nLDL or oxLDL and adjusted to 200 ng protein/10^4^ cells for nLDL, oxLDL, or phosphate buffer (50 mM PBS, pH 7.4) in the same volume as LDL for the control group. Subsequently, the cells were incubated at 2 mL/dish at 37 °C and 5% CO_2_ for 24 h. At the end of the stimulation, the cell supernatant was discarded. The staining solution was added at 1 mL/dish at 37 °C, 5% CO_2_, and light-shielded conditions for 30 min. The staining solution was a mixture of Clear MEM, SRfluor (a fluorescence probe for neutral lipids, Molecular Targeting Technologies, Inc., West Chester, PA, USA), Liperfluo (a fluorescence probe for lipid peroxides, Dojin Chemical Laboratories, Kumamoto, Japan), and Hoechst 33342 (a fluorescence probe for nuclei, Fujifilm Wako Pure Chemical Corporation) at a ratio of 1000:5:5:1. After staining, the cells were washed twice with PBS, and the supernatant was replaced with 2 mL/dish of FluoroBrite™ DMEM (Thermo Fisher Scientific Inc., Waltham, MA, USA). Cells were observed under a fluorescence microscope (HS all-in-one fluorescence microscope BZ-9000, Keyence Co. Ltd., Osaka, Japan). Excitation/emission wavelengths for SRfluor, Liperfluo, and Hoechst 33342 were 620 nm/700 nm, 470 nm/525 nm, and 360 nm/460 nm, respectively. Exposure times for SRfluor, Liperfluo, Hoechst 33342, and bright field observations were unified at 1/1.5, 1/2.5, 1/12, and 1/120 s, respectively.

With reference to Piao et al. [41], the images were converted to images suitable for analysis using the HS all-in-one fluorescence microscope BZ-II analysis application (Keyence Co. Ltd., Osaka, Japan) and analyzed for the total number and area of lipid droplets using ImageJ software (NIH, Bethesda, MD, USA) [41,42]. For simplicity, cells in which the entire cell could be identified were included in the analysis (number of counted cells = 53–84 in each group) (Supplementary Figure S13). The images were then converted to 8-bit grayscale images for binarization. A grayscale threshold (0–10) was applied to the images to remove hepatocellular structures that did not exhibit lipid droplet features. All particles with a circularity of 0.00–1.00 and an area of 0.1–50 µm^2^ were counted. The total number of particles was calculated using this method. The settings of the various analysis parameters were standardized for all images.

### 4.7. Real-Time PCR

To confirm the hepatocellular lipid metabolic changes induced by LDL, transcriptional expression analysis was performed under the same conditions as in LC-MS/MS experiments (*n* = 6–8 for each group) [43]. After stimulation for 24 h in an incubator, the cells were washed with PBS, and RNA was extracted from the cells using an RNA extraction kit (PureLink™ RNA Mini Kit, Thermo Fisher Scientific Inc., Waltham, MA, USA) according to the manufacturer’s instructions. RNA concentrations were measured using NanoDrop One (Thermo Fisher Scientific Inc., Waltham, MA, USA). RNA was converted to complementary DNA (cDNA) using ReverTra Ace^®^ qPCR RT Master Mix with gDNA Remover (TOYOBO, Co., Ltd., Osaka, Japan) in a thermal cycler (GeneAmp^®^ PCR System 9700, Applied Biosystems, Foster City, CA, USA). The cDNA was stored at −80 °C. For the PCR reaction, Thunderbird^®^ Next SYBER^®^ qPCR Mix (TOYOBO, Co., Ltd., Osaka, Japan) was mixed with cDNA samples and primers according to the manufacturer’s instructions. Target genes included *SOAT1*, *LIPE*, *DGAT1*, *ATGL*, *SREBP1*, *FASN*, *SCD1,* and *CAT,* and glyceraldehyde-3-phosphate dehydrogenase (*GAPDH*) was the housekeeping gene. Gene-specific primers were used to analyze gene expression (Supplementary Table S6). Gene expression levels were analyzed by the 2^−(ΔΔCq)^ method using a real-time PCR analysis system (CFX Connect Real-Times System, Bio-Rad Laboratories, Inc., Hercules, CA, USA). The expression levels of each target gene were corrected for the expression level of the housekeeping gene *GAPDH*.

### 4.8. Statistical Analysis

All data obtained were subjected to statistical analysis using the GraphPad Prism V7.0 software (GraphPad Software Inc., San Diego, CA, USA). Statistical analysis was performed using rejection tests to identify outliers where necessary. Then, we performed one-way analysis of variance (ANOVA), followed by Tukey’s multiple comparisons test, or one-way ANOVA, followed by Dunnett’s multiple comparisons test, or followed by Kruskal-Wallis test, or Student’s *t*-test. The significance level was set at 5%. All results are expressed as mean ± standard deviation (SD) or box plots.

### 4.9. Ethics Approval

Ethical approval for blood sampling from healthy subjects was obtained from Hokkaido University (approval number: 19-107-3). Informed consent was obtained from all the subjects.

## 5. Conclusions

The present study demonstrated that nLDL causes the accumulation of CE and the formation of lipid droplets, possibly due to the reduced expression of *LIPE* in hepatocytes. In contrast, oxLDL appeared to increase lipid hydroperoxide-rich LDs and PC-OOH/PC, mainly CE-OOH and PC-OOH derived from oxLDL. These results suggest that stimulation by oxLDL mediates oxidative stress in the liver and could trigger NASH development. The limitation of the present study is that it is difficult to determine whether this model is closer to NASH or NAFLD because it is a simple cell experiment. Further studies using various cell lines, primary cells, and in vivo experiments are needed to determine the interaction between oxLDL and the liver in detail. However, our demonstration of an association between oxLDL and hepatocytes may lead to new findings in understanding the pathogenicity of oxLDL in NASH. In addition, our data suggest that oxLDL could be established as a novel pharmacological target and candidate biomarker for NAFLD/NASH.

## Data Availability

Not applicable.

## Supplementary Materials

The following supporting information can be downloaded at https://www.mdpi.com/article/10.3390/ijms24054281/s1.
