# Supplementary material for: Oxidized Low-Density Lipoproteins Trigger Hepatocellular Oxidative Stress with the Formation of Cholesteryl Ester Hydroperoxide-Enriched Lipid Droplets
Source: Int J Mol Sci. 2023 Feb 21;24(5):4281. doi: 10.3390/ijms24054281 (PMC10002183; doi:10.3390/ijms24054281)
Supplement: Supplementary file 1 [file ijms-24-04281-s001.zip › ijms-2188988-supplementary.pdf]

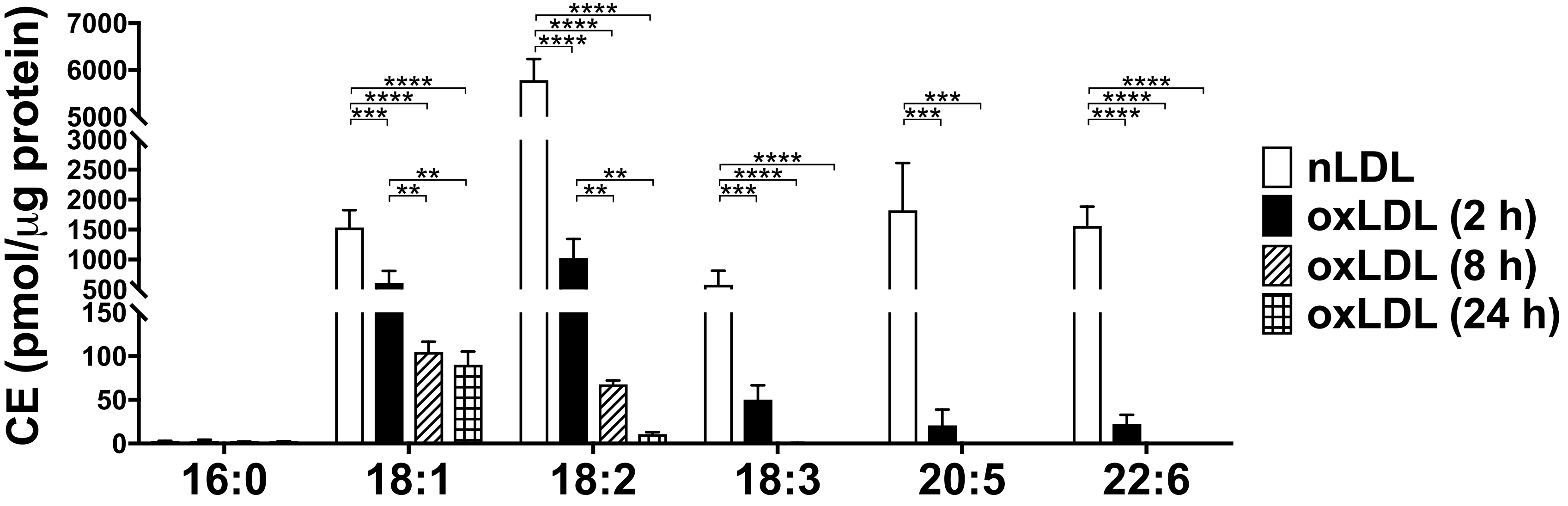

Supplementary Figure S1. Comparison of CE species in the LDL detected using Orbitrap LC-MS/MS. Results are shown as mean ± standard deviation. n = 4. One-way analysis of variance (ANOVA) with Tukey's multiple comparisons test, ** $p < 0.01$, *** $p < 0.001$, **** $p < 0.0001$. CE 20:5 was not detected in oxLDL (24 h).

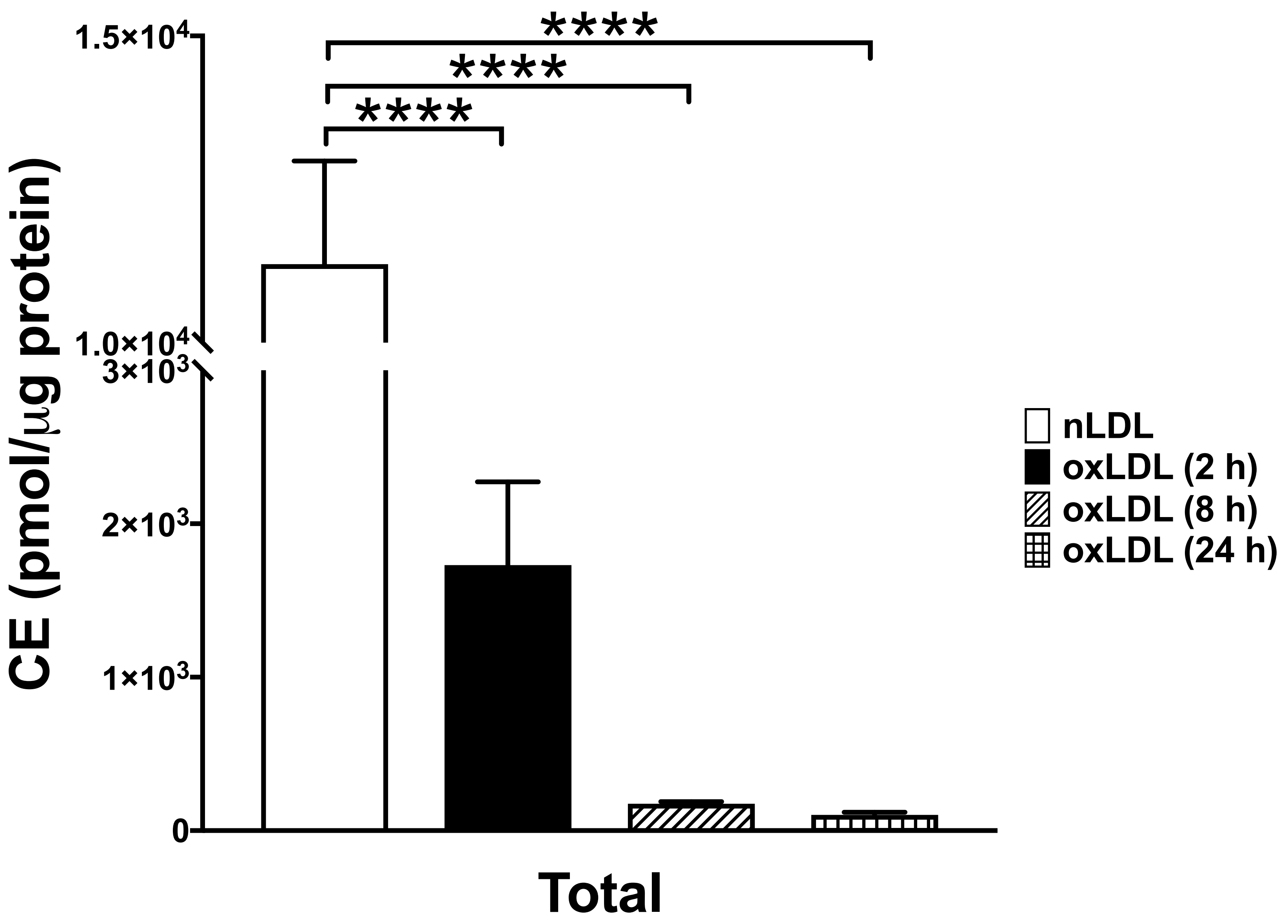

Supplementary Figure S2. Comparison of the sum of CE species in the LDL detected using Orbitrap LC-MS/MS. Results are shown as mean ± standard deviation. n = 4. One-way ANOVA with Tukey's multiple comparisons test, **** $p < 0.0001$.

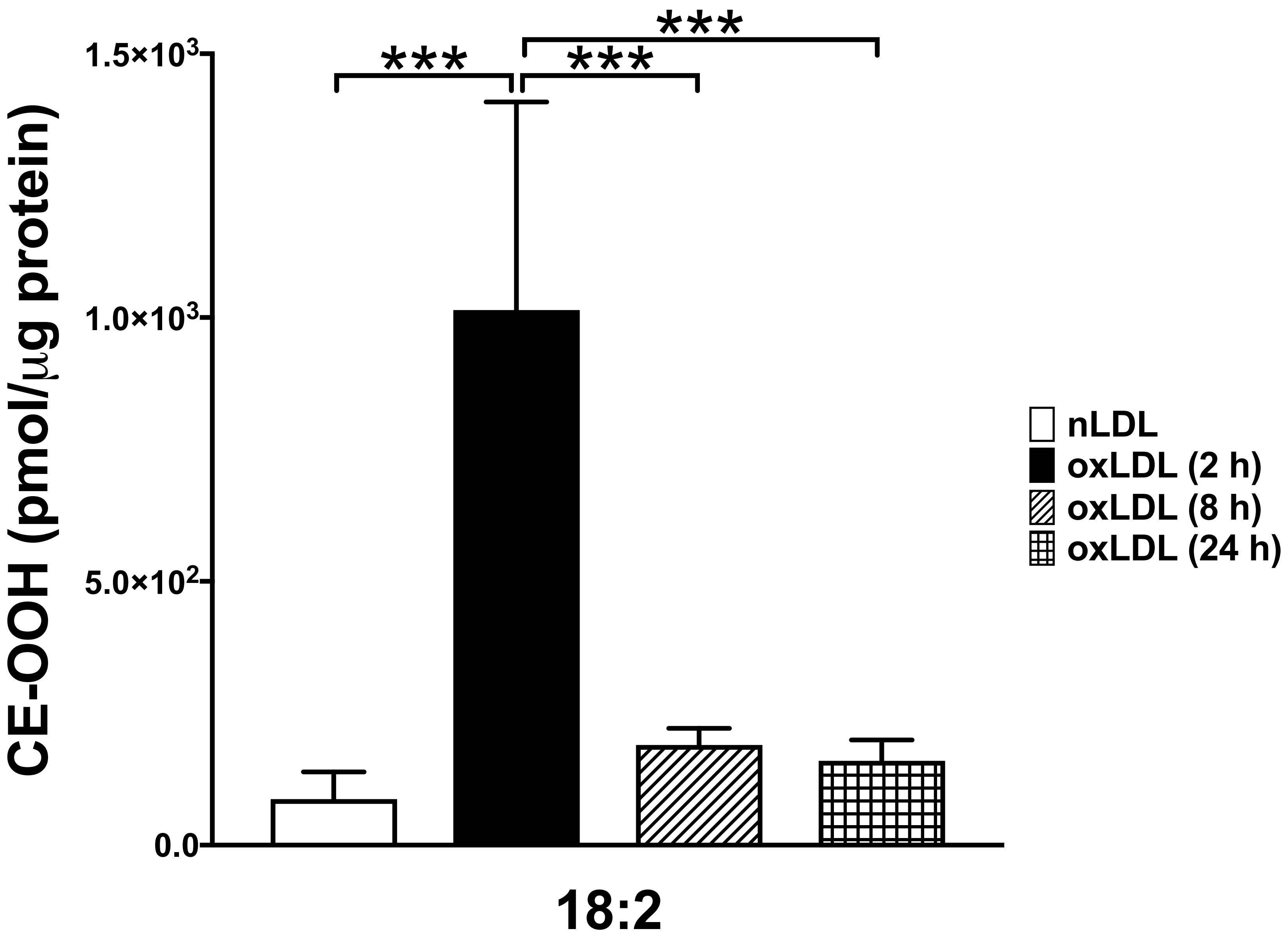

Supplementary Figure S3. Comparison of CE-OOH species in the LDL detected using Orbitrap LC-MS/MS. Results are shown as mean ± standard deviation. n = 4. One-way ANOVA with Tukey's multiple comparisons test, *** $p < 0.001$.

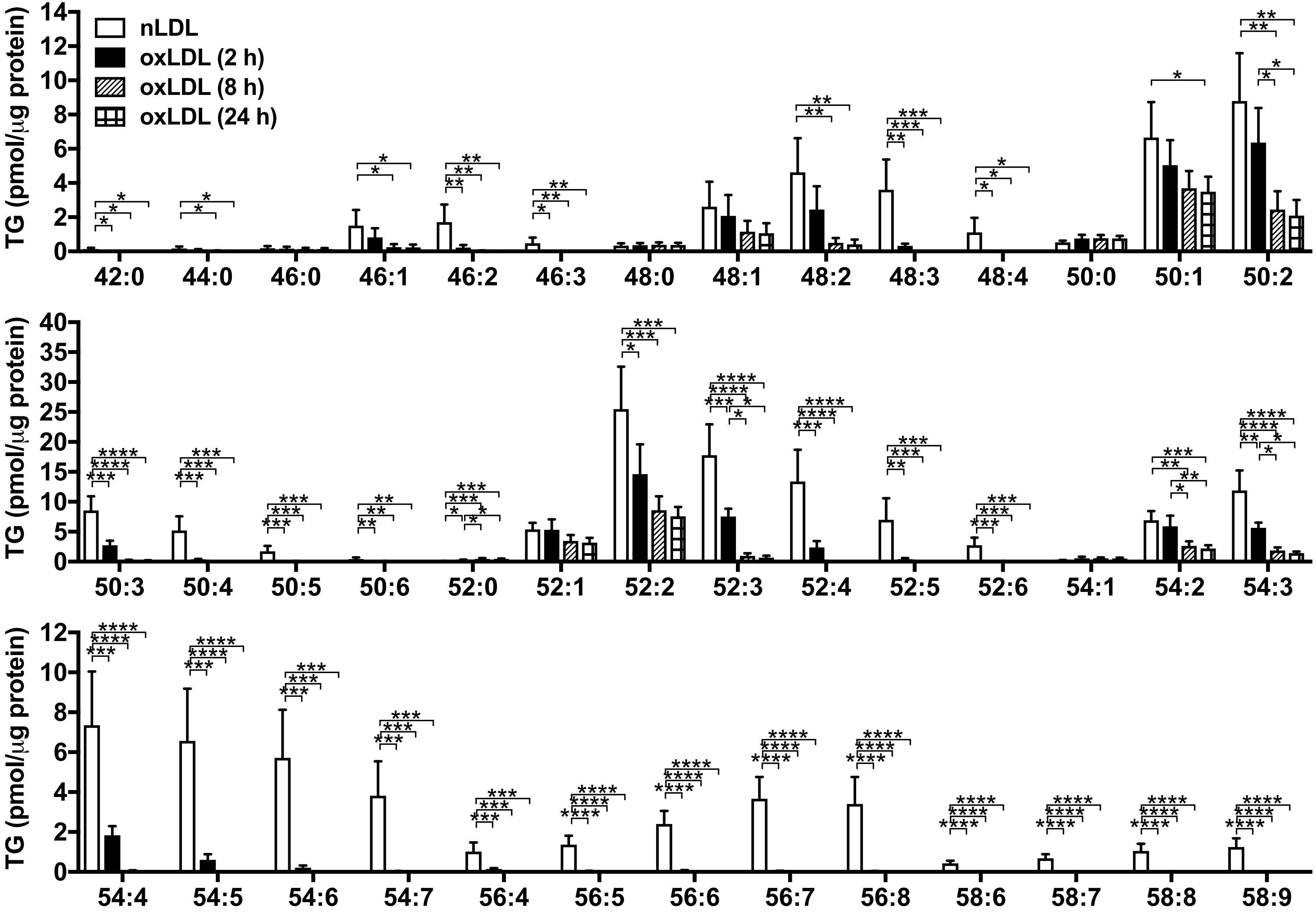

Supplementary Figure S4. Comparison of TG species in the LDL detected using Orbitrap LC-MS/MS. Results are shown as mean ± standard deviation. n = 4. One-way ANOVA with Tukey's multiple comparisons test, * $p < 0.05$, ** $p < 0.01$, *** $p < 0.001$, **** $p < 0.0001$.

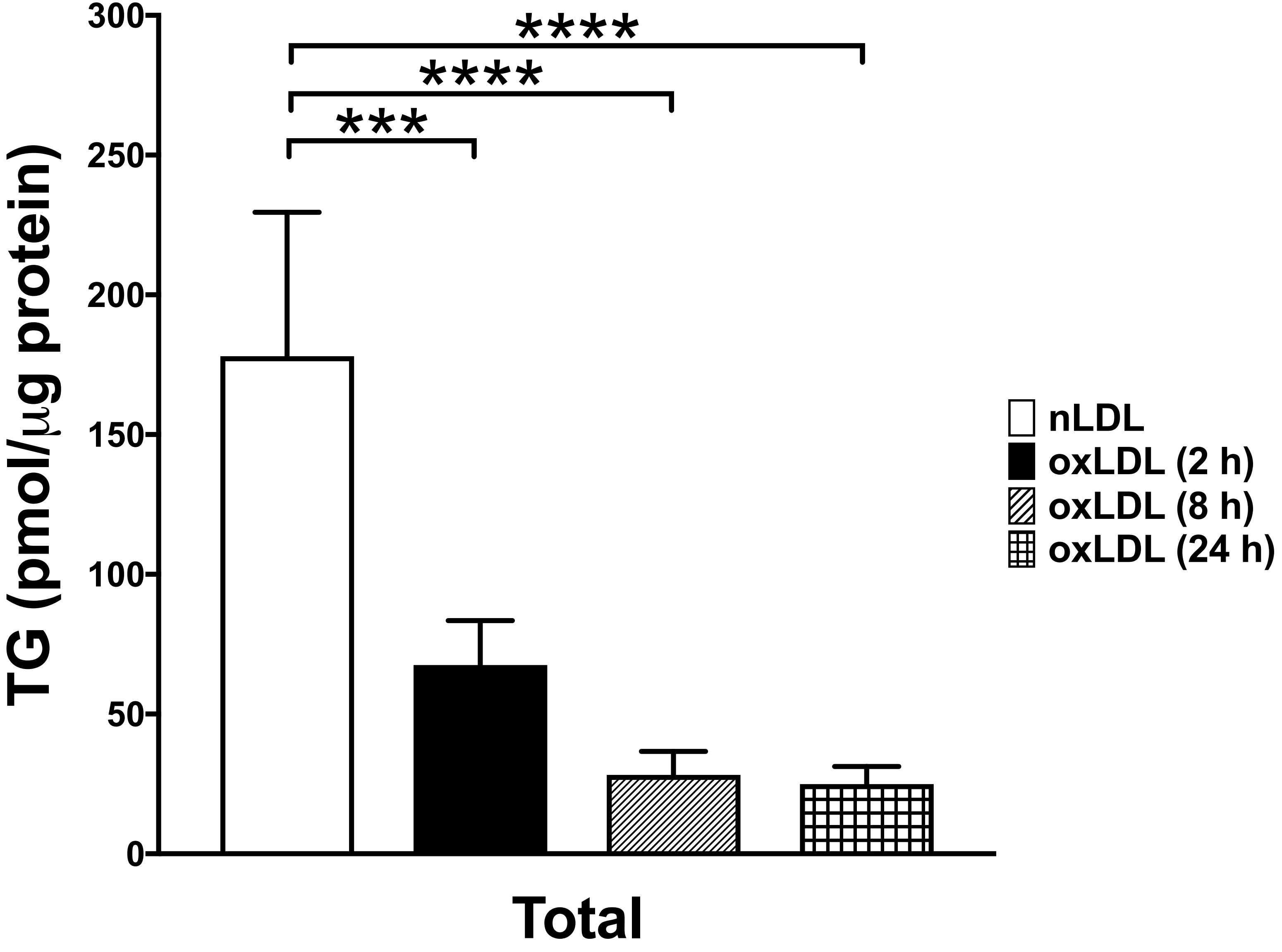

Supplementary Figure S5. Comparison of the sum of TG species in the LDL detected using Orbitrap LC-MS/MS. Results are shown as mean ± standard deviation. n = 4. One-way ANOVA with Tukey's multiple comparisons test, *** $p < 0.001$, **** $p < 0.0001$.

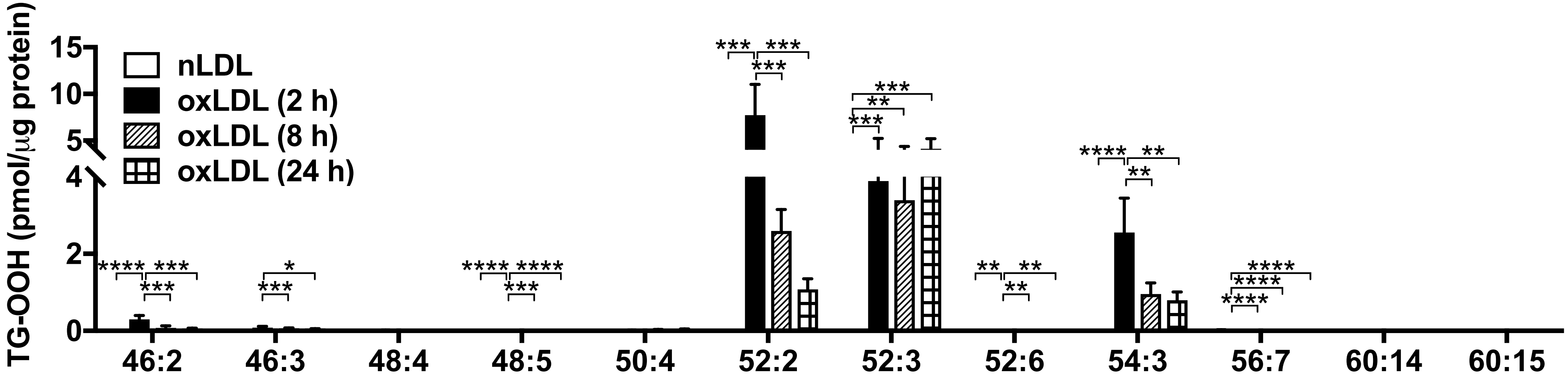

Supplementary Figure S6. Comparison of TG-OOH species in the LDL detected using Orbitrap LC-MS/MS. Results are shown as mean ± standard deviation. n = 4. One-way ANOVA with Tukey's multiple comparisons test, * $p < 0.05$, ** $p < 0.01$, *** $p < 0.001$, **** $p < 0.0001$. TG-OOH 60:14 was not detected in oxLDL (2, 8, 24 h).

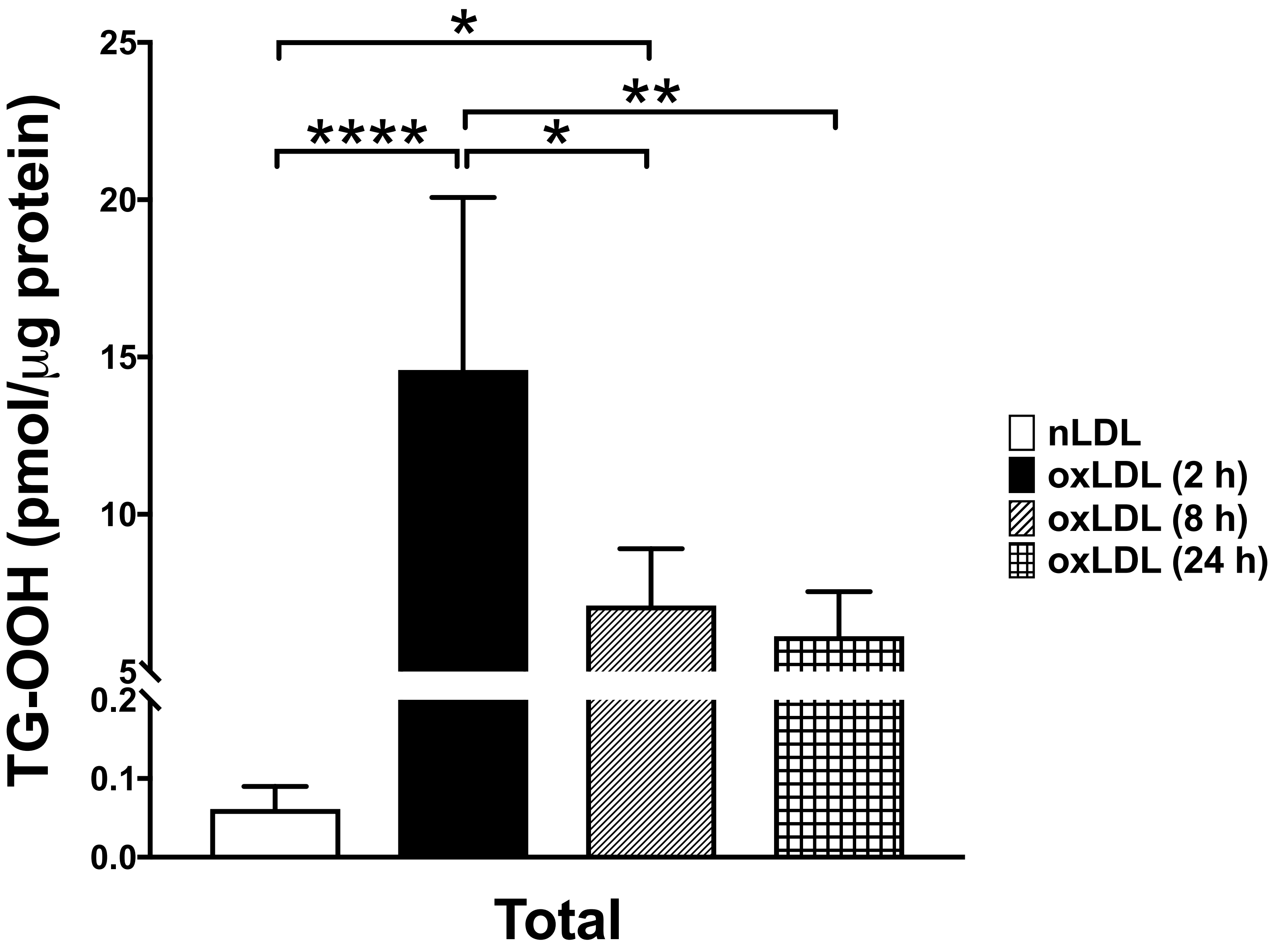

Supplementary Figure S7. Comparison of the sum of TG-OOH species in the LDL detected using Orbitrap LC-MS/MS. Results are shown as mean ± standard deviation. n = 4. One-way ANOVA with Tukey's multiple comparisons test, * $p < 0.05$, ** $p < 0.01$, **** $p < 0.0001$.

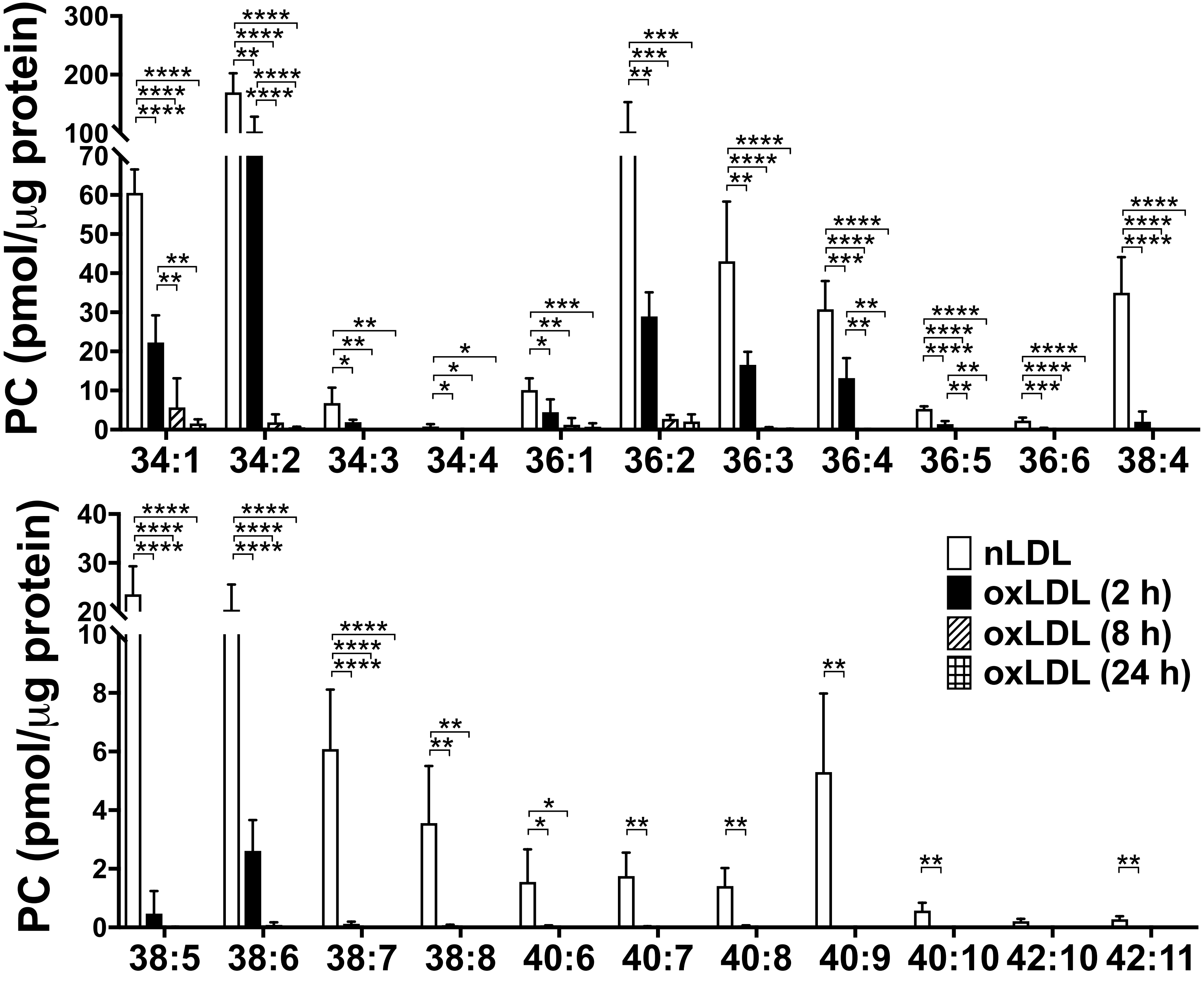

Supplementary Figure S8. Comparison of PC species in the LDL detected using Orbitrap LC-MS/MS. Results are shown as mean ± standard deviation. n = 4. One-way ANOVA with Tukey's multiple comparisons test or Student's t-test, * $p < 0.05$, ** $p < 0.01$, *** $p < 0.001$, **** $p < 0.0001$. PC 38:8 and 40:6 were undetected in oxLDL (24 h) only. PC 40:7, 40:8, 40:9, 40:10, and 42:11 were undetected in oxLDL (8 and 24 h). PC 42:10 was undetected in oxLDL (2, 8, and 24 h).

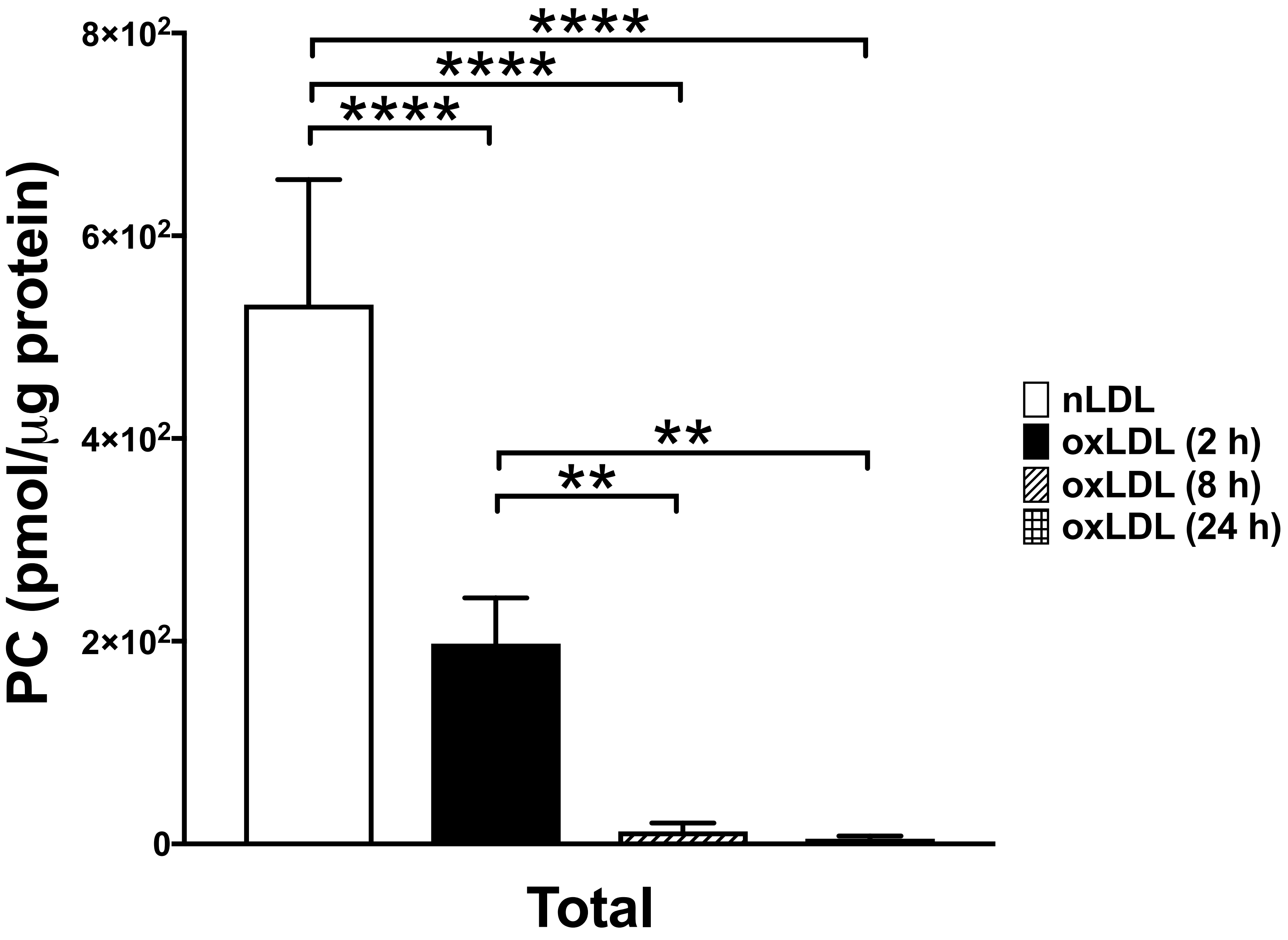

Supplementary Figure S9. Comparison of the sum of PC species in the LDL detected using Orbitrap LC-MS/MS. Results are shown as mean ± standard deviation. n = 4. One-way ANOVA with Tukey's multiple comparisons test, ** $p < 0.01$, **** $p < 0.0001$.

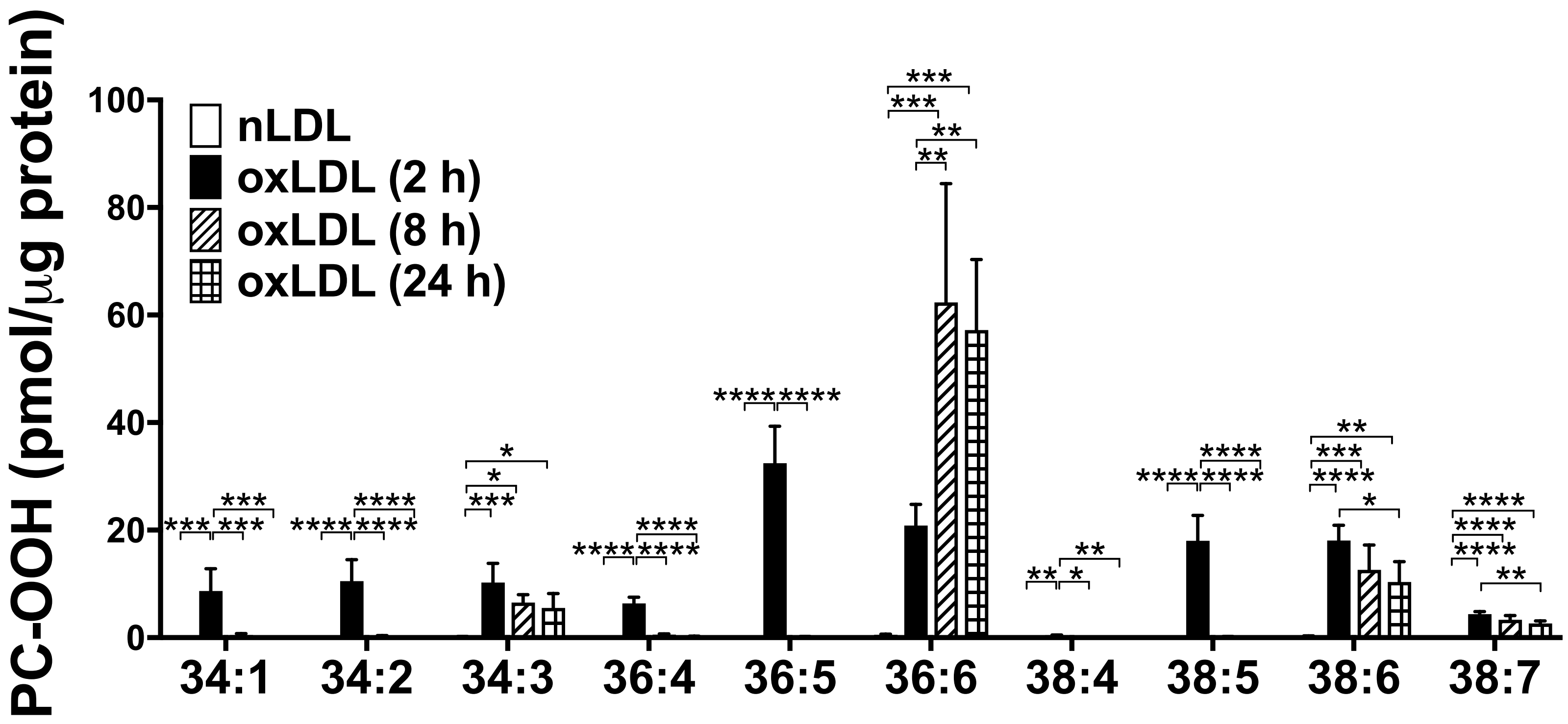

Supplementary Figure S10. Comparison of PC-OOH species in the LDL detected using Orbitrap LC-MS/MS. Results are shown as mean ± standard deviation. n = 4. One-way ANOVA with Tukey's multiple comparisons test, * $p < 0.05$, ** $p < 0.01$, *** $p < 0.001$, **** $p < 0.0001$. PC-OOH 36:5 was undetected in oxLDL (24 h) only.

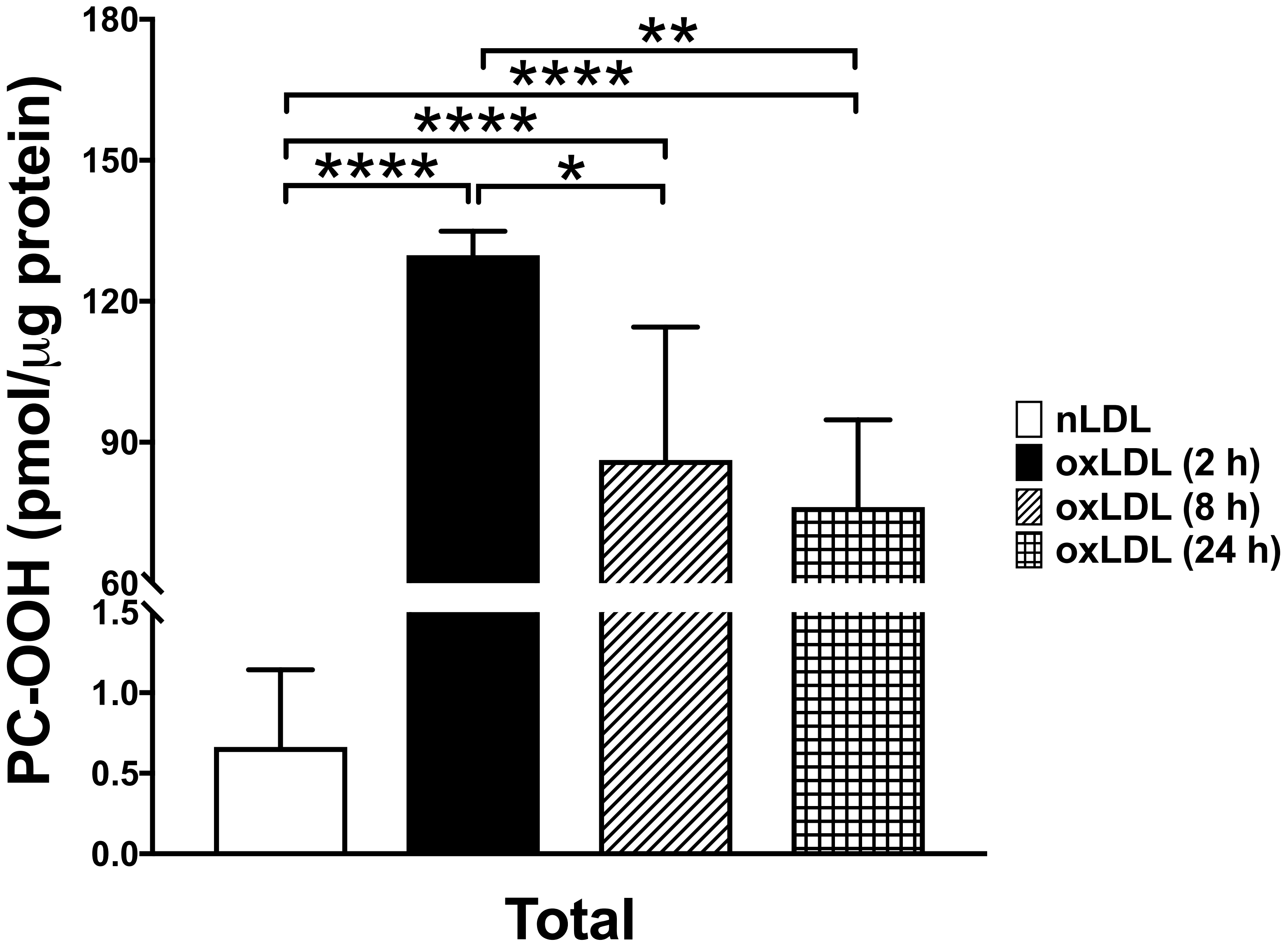

Supplementary Figure S11. Comparison of the sum of PC-OOH species in the LDL detected using Orbitrap LC-MS/MS. Results are shown as mean ± standard deviation. n = 4. One-way ANOVA with Tukey's multiple comparisons test, * $p < 0.05$, ** $p < 0.01$, **** $p < 0.0001$.

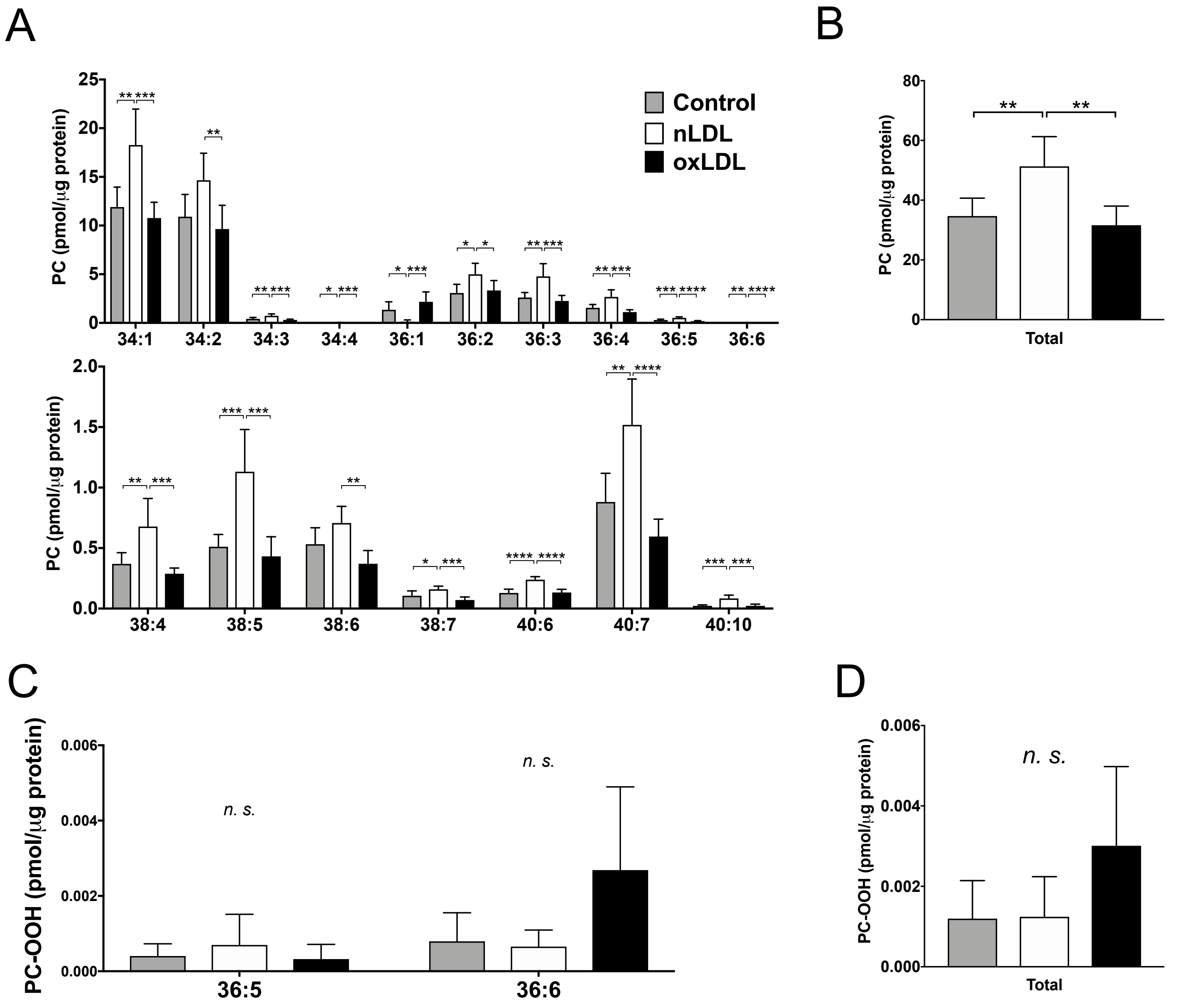

Supplementary Figure S12. Comparison of PC and PC-OOH species in the LDL-supplemented C3A cells detected using Orbitrap LC-MS/MS. (A) PC species, (B) the sum of PCs detected in this experiment, (C) PC-OOH species, and (D) the sum of PC-OOHs detected in this experiment. Results are shown as mean ± standard deviation. n = 5–6. One-way ANOVA with Tukey's multiple comparisons test, * $p < 0.05$, ** $p < 0.01$, *** $p < 0.001$, **** $p < 0.0001$, *n.s.* not significant.

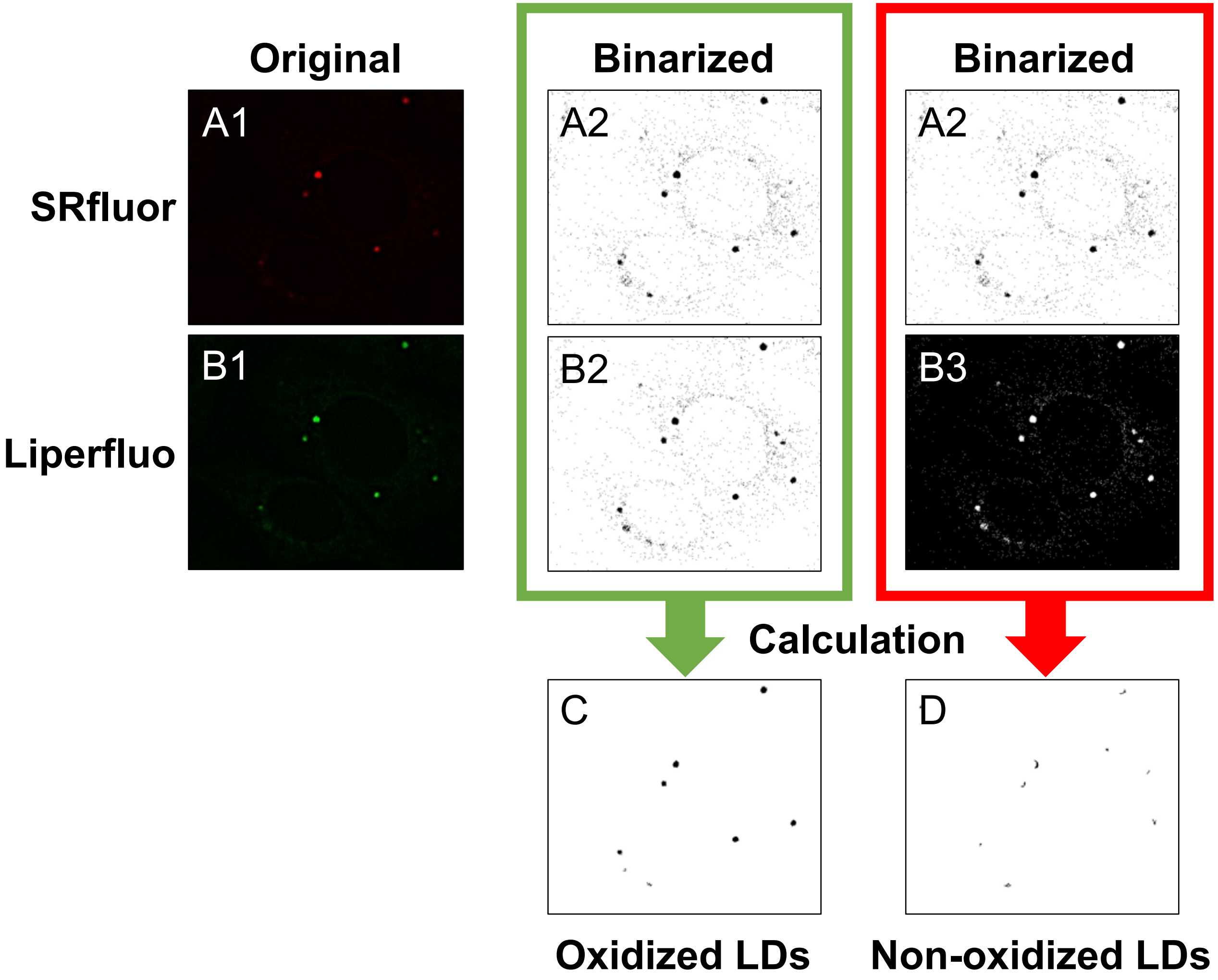

Supplementary Figure S13. Scheme of the protocol for fluorescence image analysis. Original images (A1 and B1) were binarized (A2 and B2). The black area means positive area of each fluorescence. To acquire an intersectional image, a common black area for SRfluor positive (A2) and Liperfluo positive (B2) was obtained by using an image calculator, ImageJ. An intersectional image was shown as the oxidized LDs (oxLDs) in image (C). B3 was inverted from B2 and shown as Liperfluo negative area. To acquire an intersectional image, a common black area for SRfluor positive (A2) and Liperfluo negative (B3) was obtained. An intersectional image was shown as the non-oxidized LDs (non-oxLDs) in image (D). C and D were analyzed for the number of black areas by ImageJ and shown as the number of oxLDs and non-oxLDs per cell.

Supplementary Table S1. Gradient program for electrospray ionization positive ion mode.

Mobile phase, %	Time (min)					
	0	1	10	27	27.5	30
A (5 mM aqueous ammonium acetate)	4	4	2	2	4	4
B (Isopropanol)	6	6	83	83	6	6
C (Methanol)	90	90	15	15	90	90

Supplementary Table S2. Identification of intact lipids detected in C3A cells.

Lipid species	RT (min)	Ion	Calc. (m/z)	Test (m/z)	Δppm	MS/MS signals	Molecule species
CE							
18:1	16.12	$[M+NH_4]^+$	668.6340	668.6334	-0.90		
18:2	15.80	$[M+NH_4]^+$	666.6184	666.6179	-0.75		
18:3	15.42	$[M+NH_4]^+$	664.6027	664.6022	-0.75		
20:5	15.20	$[M+NH_4]^+$	688.6027	688.6035	1.16		
22:6	15.40	$[M+NH_4]^+$	714.6184	714.6180	-0.56		
TG							
42:0	13.60	$[M+NH_4]^+$	740.6763	740.6766	0.41	-	
44:0	13.99	$[M+NH_4]^+$	768.7076	768.7086	1.30	-	
46:0	14.38	$[M+NH_4]^+$	796.7389	796.7401	1.51	-	
46:1	14.06	$[M+NH_4]^+$	794.7232	794.7235	0.38	549, 523, 521	14:0/16:1/16:0
46:2	13.72	$[M+NH_4]^+$	792.7076	792.7076	0.00	-	
46:3	13.41	$[M+NH_4]^+$	790.6919	790.6906	-1.64	-	
48:0	14.77	$[M+NH_4]^+$	824.7702	824.7715	1.58	-	
48:1	14.40	$[M+NH_4]^+$	822.7545	822.7564	2.31	-	
48:2	14.10	$[M+NH_4]^+$	820.7389	820.7389	0.00	547, 549<br>575, 521, 549	16:0/16:1/16:1<br>14:0/18:1/16:1
48:3	13.80	$[M+NH_4]^+$	818.7232	818.7239	0.85	-	

48:4	13.54	$[M+NH_4]^+$	816.7076	816.7069	-0.86	-	
50:0	15.18	$[M+NH_4]^+$	852.8015	852.8021	0.70	-	
50:1	14.81	$[M+NH_4]^+$	850.7858	850.7869	1.29	577, 551	16:0/18:1/16:0
50:2	14.48	$[M+NH_4]^+$	848.7702	848.7698	-0.47	575, 549, 577	16:0/18:1/16:1
50:3	14.19	$[M+NH_4]^+$	846.7545	846.7535	-1.18	575, 547	16:1/18:1/16:1
50:4	13.93	$[M+NH_4]^+$	844.7389	844.7388	-0.12	-	
52:0	15.60	$[M+NH_4]^+$	880.8328	880.8334	0.68	-	
52:1	15.20	$[M+NH_4]^+$	878.8171	878.8178	0.80	605, 579, 577	16:0/18:1/18:0
52:2	14.86	$[M+NH_4]^+$	876.8015	876.8019	0.46	603, 577	16:0/18:1/18:1
52:3	14.55	$[M+NH_4]^+$	874.7858	874.7858	0.00	603, 575<br>601, 577, 575	16:1/18:1/18:1<br>16:0/18:2/18:1
52:4	14.30	$[M+NH_4]^+$	872.7702	872.7706	0.46	601, 575, 573	16:1/18:2/18:1
54:0	16.05	$[M+NH_4]^+$	908.8641	908.8649	0.88	-	
54:1	15.63	$[M+NH_4]^+$	906.8484	906.8481	-0.33	605, 607<br>633, 607, 577	18:0/18:1/18:0<br>16:0/18:1/20:0
54:2	15.26	$[M+NH_4]^+$	904.8328	904.8328	0.00	603, 605<br>631, 605, 577	18:0/18:1/18:1<br>16:0/18:1/20:1
54:3	14.91	$[M+NH_4]^+$	902.8171	902.8162	-1.00	603<br>629, 603, 577	18:1/18:1/18:1<br>16:0/18:1/20:2
54:4	14.66	$[M+NH_4]^+$	900.8015	900.8015	0.00	601, 603<br>627, 601, 577	18:1/18:2/18:1<br>16:0/18:1/20:3
56:4	15.03	$[M+NH_4]^+$	928.8328	928.8321	-0.75	-	
56:5	14.77	$[M+NH_4]^+$	926.8171	926.8175	0.43	-	

PC							
34:1	10.32	$[M+H]^+$	760.5851	760.5849	-0.26	504, 478	16:0/18:1
34:2	9.50	$[M+H]^+$	758.5694	758.5695	0.13	504, 494, 476<br>502, 496, 478	16:1/18:1<br>16:0/18:2
34:3	9.16	$[M+H]^+$	756.5538	756.5534	-0.53	-	
34:4	8.92	$[M+H]^+$	754.5381	754.5367	-1.86	-	
36:1	12.15	$[M+H]^+$	788.6164	788.6156	-1.01	504, 506	18:0/18:1
36:2	10.77	$[M+H]^+$	786.6007	786.6005	-0.25	504<br>502	18:1/18:1<br>18:0/18:2
36:3	10.05	$[M+H]^+$	784.5851	784.5846	-0.64	-	
36:4	9.49	$[M+H]^+$	782.5694	782.5685	-1.15	-	
36:5	9.03	$[M+H]^+$	780.5538	780.5528	-1.28	-	
36:6	8.62	$[M+H]^+$	778.5381	778.5387	0.77	-	
38:4	10.11	$[M+H]^+$	810.6007	810.6005	-0.25	-	
38:5	9.58	$[M+H]^+$	808.5851	808.5837	-1.73	-	
38:6	9.30	$[M+H]^+$	806.5694	806.5682	-1.49	-	
38:7	8.94	$[M+H]^+$	804.5538	804.5530	-0.99	-	
40:6	10.08	$[M+H]^+$	834.6007	834.6010	0.36	-	
40:7	9.50	$[M+H]^+$	832.5851	832.5842	-1.08	-	
40:10	8.99	$[M+H]^+$	826.5381	826.5363	-2.18	-	

-: not available.

Supplementary Table S3. Identification of hydroperoxides detected in C3A cells.

Lipid species	RT (min)	Ion	Calc. (m/z)	Test (m/z)	Δppm
CE-OOH					
18:1	12.86	$[M+NH_4]^+$	700.6238	700.6213	-3.57
18:2	12.45	$[M+NH_4]^+$	698.6082	698.6079	-0.43
22:6	12.17	$[M+NH_4]^+$	746.6082	746.6064	-2.41
TG-OOH					
52:2	13.28	$[M+NH_4]^+$	908.7913	908.7943	3.30
56:7	12.40	$[M+NH_4]^+$	954.7756	954.7729	-2.83
62:12	12.59	$[M+NH_4]^+$	1028.7913	1028.7947	3.30
PC-OOH					
36:5	6.97	$[M+H]^+$	812.5436	812.5422	-1.72
36:6	6.32	$[M+H]^+$	810.5280	810.5273	-0.86

Supplementary Table S4. Identification of intact lipids detected in LDL.

Lipid species	RT (min)	Ion	Calc. (m/z)	Test (m/z)	Δppm	MS/MS signals	Molecule species
CE							
16:0	16.18	$[M+NH_4]^+$	642.6184	642.6172	-1.87		
18:1	16.20	$[M+NH_4]^+$	668.6340	668.6323	-2.54		
18:2	15.83	$[M+NH_4]^+$	666.6184	666.6180	-0.60		
18:3	15.49	$[M+NH_4]^+$	664.6027	664.6035	1.20		
20:5	15.38	$[M+NH_4]^+$	688.6027	688.6027	0.00		
22:6	15.49	$[M+NH_4]^+$	714.6184	714.6190	0.84		
TG							
42:0	13.88	$[M+NH_4]^+$	740.6763	740.6766	0.41	-	
44:0	14.28	$[M+NH_4]^+$	768.7076	768.7081	0.65	-	
46:0	14.62	$[M+NH_4]^+$	796.7389	796.7386	-0.38	-	
46:1	14.30	$[M+NH_4]^+$	794.7232	794.7238	0.75	-	
46:2	14.03	$[M+NH_4]^+$	792.7076	792.7076	0.00	-	
46:3	13.74	$[M+NH_4]^+$	790.6919	790.6912	-0.89	-	
48:0	14.96	$[M+NH_4]^+$	824.7702	824.771	0.97	-	
48:1	14.66	$[M+NH_4]^+$	822.7545	822.7552	0.85	-	
48:2	14.38	$[M+NH_4]^+$	820.7389	820.7397	0.97	547, 549<br>575, 521, 549	16:0/16:1/16:1<br>14:0/18:1/16:1

48:3	14.11	$[M+NH_4]^+$	818.7232	818.723	-0.24	547<br>573, 521, 547	16:1/16:1/16:1<br>14:0/18:2/16:1
48:4	13.85	$[M+NH_4]^+$	816.7076	816.7057	-2.33	-	
50:0	15.33	$[M+NH_4]^+$	852.8015	852.8014	-0.12	-	
50:1	15.00	$[M+NH_4]^+$	850.7858	850.7866	0.94	577, 551	16:0/18:1/16:0
50:2	14.71	$[M+NH_4]^+$	848.7702	848.7709	0.82	575, 549, 577	16:0/18:1/16:1
50:3	14.46	$[M+NH_4]^+$	846.7545	846.7549	0.47	575, 547	16:1/18:1/16:1
50:4	14.20	$[M+NH_4]^+$	844.7389	844.7389	0.00	573, 547	16:1/18:2/16:1
50:5	13.98	$[M+NH_4]^+$	842.7232	842.722	-1.42	-	
50:6	13.83	$[M+NH_4]^+$	840.7076	840.7067	-1.07	-	
52:0	15.68	$[M+NH_4]^+$	880.8328	880.8337	1.02	-	
52:1	15.38	$[M+NH_4]^+$	878.8171	878.8168	-0.34	605, 579, 577	16:0/18:1/18:0
52:2	15.04	$[M+NH_4]^+$	876.8015	876.8011	-0.46	603, 577	16:0/18:1/18:1
52:3	14.82	$[M+NH_4]^+$	874.7858	874.7859	0.11	603, 575<br>601, 577, 575	16:1/18:1/18:1<br>16:0/18:2/18:1
52:4	14.52	$[M+NH_4]^+$	872.7702	872.7697	-0.57	601, 575, 573	16:1/18:2/18:1
52:5	14.30	$[M+NH_4]^+$	870.7545	870.7549	0.46	599, 573	16:1/18:2/18:2
52:6	14.09	$[M+NH_4]^+$	868.7389	868.7384	-0.58	-	
54:1	15.74	$[M+NH_4]^+$	906.8484	906.8479	-0.55	-	
54:2	15.42	$[M+NH_4]^+$	904.8328	904.8314	-1.55	603, 605<br>631, 605, 577	18:0/18:1/18:1<br>16:0/18:1/20:1
54:3	15.13	$[M+NH_4]^+$	902.8171	902.8148	-2.55	603<br>629, 603, 577	18:1/18:1/18:1<br>16:0/18:1/20:2

54:4	14.88	$[M+NH_4]^+$	900.8015	900.7995	-2.22	601, 603<br>627, 601, 577	18:1/18:2/18:1<br>16:0/18:1/20:3
54:5	14.64	$[M+NH_4]^+$	898.7858	898.7841	-1.89	599, 601<br>625, 601, 575	18:1/18:2/18:2<br>16:0/18:2/20:3
54:6	14.46	$[M+NH_4]^+$	896.7702	896.7687	-1.67	-	
54:7	14.25	$[M+NH_4]^+$	894.7545	894.754	-0.56	-	
56:4	15.28	$[M+NH_4]^+$	928.8328	928.8293	-3.77	-	
56:5	15.11	$[M+NH_4]^+$	926.8171	926.8141	-3.24	-	
56:6	14.84	$[M+NH_4]^+$	924.8015	924.7984	-3.35	-	
56:7	14.64	$[M+NH_4]^+$	922.7858	922.7837	-2.28	-	
56:8	14.41	$[M+NH_4]^+$	920.7702	920.7689	-1.41	647, 623, 575	16:0/18:2/22:6
58:6	15.26	$[M+NH_4]^+$	952.8328	952.831	-1.89	-	
58:7	14.93	$[M+NH_4]^+$	950.8171	950.8152	-2.00	-	
58:8	14.71	$[M+NH_4]^+$	948.8015	948.7999	-1.69	-	
58:9	14.48	$[M+NH_4]^+$	946.7858	946.7845	-1.37	-	
PC							
34:1	10.25	$[M+H]^+$	760.5851	760.5847	-0.53	504, 478	16:0/18:1
34:2	9.74	$[M+H]^+$	758.5694	758.5692	-0.26	502, 496, 478	16:0/18:2
34:3	9.25	$[M+H]^+$	756.5538	756.5533	-0.66	-	
34:4	9.04	$[M+H]^+$	754.5381	754.5385	0.53	-	

36:1	11.94	$[M+H]^+$	788.6164	788.6140	-3.04	504, 506	18:0/18:1
36:2	10.80	$[M+H]^+$	786.6007	786.6002	-0.64	502, 506	18:0/18:2
36:3	10.01	$[M+H]^+$	784.5851	784.5826	-3.19	502, 504	18:1/18:2
36:4	9.74	$[M+H]^+$	782.5694	782.5668	-3.32	502<br>544, 526, 496, 478	18:2/18:2<br>16:0/20:4
36:5	9.54	$[M+H]^+$	780.5538	780.5508	-3.84	500, 502<br>524, 478	18:2/18:3<br>16:0/20:5
36:6	9.21	$[M+H]^+$	778.5381	778.5363	-2.31	-	
38:4	10.44	$[M+H]^+$	810.6007	810.5982	-3.08	544, 526, 524, 506	18:0/20:4
38:5	9.93	$[M+H]^+$	808.5851	808.5813	-4.70	544, 526, 522, 504	18:1/20:4
38:6	9.74	$[M+H]^+$	806.5694	806.5667	-3.35	568, 550, 496, 478	16:0/22:6
38:7	9.35	$[M+H]^+$	804.5538	804.5511	-3.36	-	
38:8	9.17	$[M+H]^+$	802.5381	802.5355	-3.24	-	
40:6	11.24	$[M+H]^+$	834.6007	834.5978	-3.47	-	
40:7	10.32	$[M+H]^+$	832.5851	832.5815	-4.32	-	
40:8	9.95	$[M+H]^+$	830.5694	830.5654	-4.82	-	
40:9	9.31	$[M+H]^+$	828.5538	828.5505	-3.98	-	
40:10	9.02	$[M+H]^+$	826.5381	826.5372	-1.09	-	
42:10	9.06	$[M+H]^+$	854.5694	854.5705	1.29	-	
42:11	9.17	$[M+H]^+$	852.5538	852.5516	-2.58	-	

-: not available.

Supplementary Table S5. Identification of hydroperoxides detected in LDL.

Lipid species	RT (min)	Ion	Calc. (m/z)	Test (m/z)	Δppm
CE-OOH					
18:1	12.86	$[M+NH_4]^+$	700.6238	700.6213	-3.57
18:2	12.45	$[M+NH_4]^+$	698.6082	698.6079	-0.43
22:6	12.17	$[M+NH_4]^+$	746.6082	746.6064	-2.41
TG-OOH					
52:2	13.28	$[M+NH_4]^+$	908.7913	908.7943	3.30
56:7	12.40	$[M+NH_4]^+$	954.7756	954.7729	-2.83
62:12	12.59	$[M+NH_4]^+$	1028.7913	1028.7947	3.30
PC-OOH					
36:5	6.97	$[M+H]^+$	812.5436	812.5422	-1.72
36:6	6.32	$[M+H]^+$	810.5280	810.5273	-0.86

Supplementary Table S6. Sequences of primers used in this study.

Gene	Forward primer (5'-3')	Reverse primer (3'-5')
SOAT1	GAAACCGGCTGTCAAAGTCC	AATGGCTTCAATTCCTCTGC
LIPE	CTCAGTGTGCTCTCCAAGTG	CACCCAGGCGGAAGTCTC
DGAT1	TATTGCGGCCAATGTCTTTGC	CACTGGAGTGATAGACTCAACCA
ATGL	ACCAGCATCCAGTTCAACCT	ATCCCTGCTTGCACATCTCT
SREBP1	CAGCCCACTTCATCAAGG	ACTGTTGCCAAGATGGTTCCG
SCD1	GACGATGAGCTCCTGCTGTT	CTCTGCTACACTTGGGAGCC
FASN	AACTCCTGCAAGTTCTCCGA	GCTCCAGCCTCGCTCTC
CAT	TTTCCCAGGAAGATCCTGAC	ACCTTGGTGAGATCGAATGG
GAPDH	GAAGGTGAAGGTCGGAGTC	GAAGATGGTGATGGGATTTC
